# The landscape of antibody production systems: recombinant expression for research, diagnostics and therapy

**DOI:** 10.3389/fbioe.2026.1765764

**Published:** 2026-05-20

**Authors:** Esther Veronika Wenzel, Laila Al-Halabi-Frenzel, Seyhan Demiral, André Frenzel, Dianne Celine Gnann, Martha Charlotte-Elisabeth Keitel, Nina Lehmler, Tarushyam Mukherjee, Justus Rauls, Thomas Schirrmann, Piotr Grzegorz Stańczyk, Stephan Steinke, Jan Terbrack, Sofie Westerhoff, Stefan Dübel, Maren Schubert, Michael Hust, Federico Bertoglio

**Affiliations:** 1 Abcalis GmbH, Braunschweig, Germany; 2 Helmholtz Centre for Infection Research, VLP-Based Technologies, Braunschweig, Germany; 3 Technische Universität Braunschweig, Institut für Biochemie, Biotechnologie und Bioinformatik, Abteilung Biotechnologie, Braunschweig, Germany; 4 Yumab GmbH, Braunschweig, Germany; 5 Technische Universität Braunschweig, Institut für Biochemie, Biotechnologie und Bioinformatik, Abteilung Medizinische Biotechnologie, Braunschweig, Germany; 6 Department of Neurology and Neuroscience, Autoimmune Neurology Group, Mayo Clinic, Jacksonville, FL, United States

**Keywords:** cell-free system, fungi, Gram negative bacteria, Gram positive bacteria, human *in vivo* production, insect cells, mammalian cells, microalgae

## Abstract

Over the past three decades, the emergence of recombinant antibodies has positioned them as the fastest-growing class of therapeutic proteins and as well as crucial reagents for both diagnostics and specific research applications. The industrial production of most therapeutic antibodies currently relies on mammalian cell lines, primarily Chinese Hamster Ovary (CHO) cells, which remain the gold standard in biopharmaceutical manufacturing due to their exceptionally high production yields, robustness, regulatory acceptance, and consistent product quality. Nevertheless, new approaches using specifically adapted expression systems are emerging. These new host organisms enable the production of antibodies with posttranslational modifications that closely resemble those found in the human system, without the need for animal-derived source materials. For diagnostic and research purposes, a broad range of production systems has been developed. These include Gram-negative and Gram-positive bacteria, yeasts, filamentous fungi, insect cell lines, and transgenic plants. Additionally, smaller single domain antibodies and bispecific fragments that do not require glycosylation can be successfully produced in bacteria for therapeutic applications. Some of these molecules have already been approved by regulatory authorities in the European Union and the United States, and several more are currently in clinical trials. This review therefore focuses on the broad and evolving landscape of recombinant antibody production systems and their applicability across various fields.

## 1 Introduction

Animal-free methods for antibody discovery, like phage display (Breitling et al., 1991; McCafferty et al., 1990; Barbas et al., 1991), were developed driven by multiple factors: the demand for fully human antibodies, practical advantages of *in vitro* discovery pipelines and ethical concerns (Dübel, 2024). These techniques are well established today to yield high quality antibodies for therapeutic, diagnostic and research use (Alfaleh et al., 2020; Zhang, 2023). Driven by quality issues and regulatory incentives for better reproducibility and batch consistency, as well as the hope for lower production cost, there has been a pronounced shift towards animal-free methods for the production of antibodies. This transition has opened the door to platforms that combine ethical benefits with speed and precision. In particular, technologies such as phage display, yeast display, ribosome display/mRNA/cell-free display systems allow the rapid isolation of high-affinity antibodies entirely *in vitro* (Breitling et al., 1991; Hoogenboom, 2005; Valldorf et al., 2022). These systems bypass the need for immunization, enabling antibody generation against non-immunogenic or toxic targets, and allow fine control over selection pressures such as affinity, stability, or epitope specificity. Naïve antibody libraries, which capture the natural diversity of the human immune repertoire without prior antigen exposure, have provided an essential foundation for rapid antibody discovery and optimization. The maturation of synthetic and semi-synthetic antibody libraries, comprising billions of fully human sequences, has been particularly transformative. Modern synthetic libraries are often designed using deep sequencing data, structural modeling, and increasingly AI-guided optimization to maximize functional diversity while maintaining developability (Aguilar Rangel et al., 2022; Tiller and Tessier, 2015).

Traditional antibody production has relied on immunization of animals, typically rodents or larger mammals, to elicit an immune response, followed by hybridoma generation or serum collection. These methods have delivered many successful antibodies for research and diagnostics, but only a few therapeutic products, which are mostly limited to one-time use due to the high immunogenicity of animal sera or murine hybridomas in patients. They are further inherently limited by species-specific immune repertoires, variable immune responses to conserved or toxic antigens and the logistical and ethical burdens of animal experimentation. Moreover, animal-derived polyclonal antibodies inherently always exhibit batch-to-batch variability and undefined specificity profiles (Hjelm et al., 2012; Li et al., 2023), complicating reproducibility in diagnostics and research.

Nevertheless, the use of animals for polyclonal antibody production for research use and diagnostic tests is still predominant. More than 50% of antibodies commercially offered for research use are still based on polyclonal sera (Dübel, 2024), despite well-known problems with false positive or lacking reactivities (Begley and Ellis, 2012; Bradbury A. M. and Plückthun, 2015; Goodman, 2018; Bradbury A. and Plückthun, 2015) and always poorly defined contents that are “littering the field with false findings” (Baker, 2015).

The use of large animals, typically sheep, goats or horses, started with generation of therapeutic antisera against diphtheria toxin and tetanus toxins (Behring and Shibasaburo, 1890), and was the only available method until 1975, when the hybridoma technique was developed (Köhler and Milstein, 1975). In addition to diphtheria antitoxin, which is still in use today, another important application of horse serum continues to be the treatment of snake venom (Pucca et al., 2019). Their use induces serum sickness and its production today is met with more and more problems (Kupferschmidt, 2020). For some antibodies so far obtained from immunized horses, animal-free production seems feasible, in particular, if the antigen is a well defined entity (Menzies et al., 2025; Wenzel et al., 2020). Polyclonal sera from small animals are rarely used for therapy, but the few drugs derived this way are still indispensable and difficult to prepare with animal-free production methods due to the very complex nature of the antigen used for immunization (whole cell preparations) and the lack of knowledge about the essential functional components within these highly diverse antibody mixes, e.g., anti-human-T-lymphocyte immunoglobulin made in rabbits (Nirmal et al., 2024). Larger quantities of monoclonal antibodies were produced using the ascites method, in which hybridomas are injected in the belly of mice, a method now banned in many countries (Jackson et al., 1999; Yokoyama, 2000).

Transgenic animals also have been employed to produce recombinant antibodies in large scale, mainly by secretion into the milk (Pollock et al., 1999) but failed to demonstrate superiority over bioreactor-based methods until today.

Consequently, besides the move to animal-free antibody discovery, another crucial development is the move toward animal-component-free biomanufacturing in antibody production. Regulatory authorities and industry increasingly favor serum-free, chemically-defined media and fully recombinant production cell lines. This shift improves product consistency, reduces contamination risks (e.g., from adventitious agents), and aligns with ethical frameworks such as the 3Rs (Replacement, Reduction, Refinement) guiding animal use in research (Weber et al., 2025).

In parallel with the expansion of antibody applications, alternative production platforms have diversified far beyond traditional mammalian cell culture systems, offering novel solutions for cost-effective, scalable, and flexible manufacturing. On the prokaryotic side, both Gram-negative bacteria, such as *Escherichia coli*, and Gram-positive hosts, including various *Bacillus* species, are widely used for the expression of antibody fragments due to their rapid growth and genetic tractability. Advances in secretion systems, periplasmic folding, and strain engineering have significantly improved yields and functionality in these hosts.

Among eukaryotic production systems, yeasts (e.g., *Komagataella phaffii* (previously named *Pichia pastoris)* and *Saccharomyces cerevisiae*) remain important platforms, particularly for antibody fragments, benefiting from eukaryotic protein processing combined with microbial robustness and rapid growth. Filamentous fungi have gained renewed interest for secreting full-length antibodies at high titers, while microalgae, including both *Chlorophyta* (green algae) and *Bacillariophyta* (diatoms), are emerging as sustainable and versatile hosts, combining eukaryotic folding machinery with low-cost cultivation. In addition, protozoa represent an unconventional but increasingly explored class of expression systems with unique post-translational capabilities.

Insect cell systems continue to mature as powerful eukaryotic platforms. Here, while baculovirus-based expression remains a well-established method for producing complex antibody formats, baculovirus-free insect cell systems are now being developed, thus enabling faster, more flexible, and regulatory-friendly manufacturing. Meanwhile, mammalian cells, such as CHO, remain the gold standard for producing therapeutic-grade antibodies, both in stable cell line systems for large-scale commercial manufacturing and transient expression setups for rapid candidate screening and early development.

Plant-based expression systems, particularly transgenic plants like *Nicotiana benthamiana*, are gaining momentum as cost-efficient and scalable platforms, aided by advances in glyco-engineering that enable human-compatible post-translational modifications. Cell-free systems are also emerging as a versatile and animal-free alternative for rapid prototyping and small- to medium-scale production, offering unique advantages in speed, automation, and biosafety. Finally, *in vivo* human antibody production approaches represent a frontier concept, leveraging human hosts themselves for antibody generation.

**FIGURE 1:**
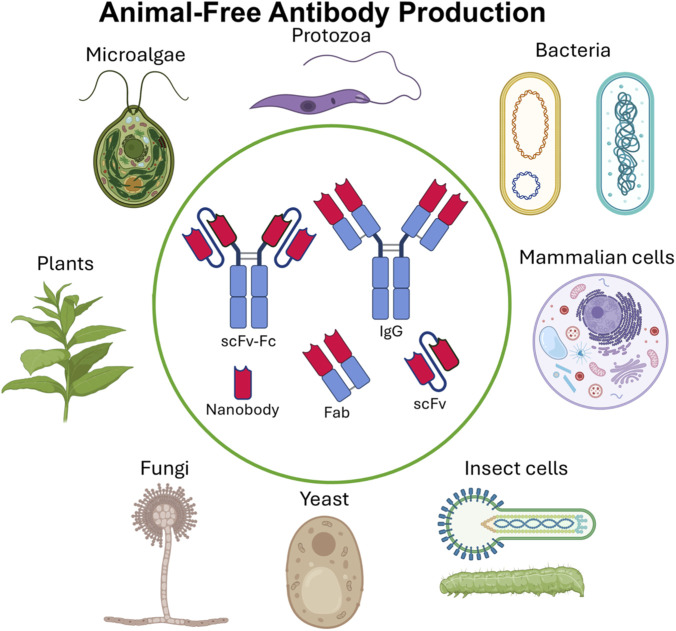
Animal-free antibody production systems for different antibody formats (Created with BioRender.com/mxkcxpn).

Together, these diverse and rapidly evolving production systems form a rich technological landscape, each with distinct advantages and challenges depending on the antibody format, required post-translational modifications, regulatory considerations, and intended application. In the following sections of this review, we will examine each of these platforms in detail, highlighting recent advances, current capabilities, and their potential roles in shaping the future of animal-free antibody production systems (Figure 1).

## 2 Eukaryotic hosts used for antibody production

### 2.1 Mammalian cells

Given that clinically-approved antibodies are almost exclusively produced in mammalian cells, we will first present these production hosts, focusing on more experimental and less employed platforms later on in the text.

Antibodies are produced by B lymphocytes and their terminally differentiated plasma cells, and this is the natural way mammals generate antigen specific humoral immunity (Fagraeus, 1947). Today, mammalian cell lines, particularly Chinese Hamster Ovary (CHO) and Human Embryonic Kidney (HEK293) cells, continue to be the preferred platforms for producing complex recombinant proteins used in therapeutics, diagnostics, and academic research. Despite higher production costs and longer process times, they are unmatched in their ability of accurate protein folding, assembly, glycosylation, and post-translational modifications in a way that closely mimics human physiology. Approximately 60%–70% of all recombinant protein pharmaceuticals and nearly all therapeutic antibodies are still produced in mammalian hosts. These same systems are now widely applied for recombinant diagnostic antigens, monoclonal antibodies, and reference standards in research and quality control (Tan et al., 2021; Li et al., 2021).

In recent years, there has been a strong shift toward animal-component-free and chemically-defined culture systems, which eliminate serum and other animal-derived components from cell culture media. Fully synthetic formulations, often supplemented with recombinant growth factors and albumin, enable high cell densities and reproducible protein production while reducing ethical concerns and risks of contamination. Modern CHO and HEK293 cell lines routinely achieve high productivity in these animal-component-free systems without compromising product quality, making them suitable for both commercial and academic applications (Weber et al., 2025).

Human-derived cell lines such as HEK293 and PER.C6 (human embryonic retinoblast cells) are increasingly employed for diagnostic proteins and transient production, where human-like post-translational modifications are essential (Lifely et al., 1995).

Common examples for animal-derived cell lines for recombinant antibody production, particularly in research and early development stages, include NS0 (murine myeloma cells) and BHK-21 (baby hamster kidney cells) (Inoue et al., 1996; Dhara et al., 2018). One significant advantage of these non-human cell lines for therapeutic purposes is their low susceptibility to human viral contamination, which enhances biosafety during manufacturing. This feature reduces the risk of introducing adventitious human pathogens into biopharmaceutical products, simplifying viral clearance validation. Here, CHO cells remain the workhorse of industrial production, supported by extensive cell-line engineering and process optimization.

However, animal-derived cell lines generally exhibit non-human glycosylation patterns, which may require additional engineering to minimize immunogenicity. Genetic and metabolic engineering through CRISPR/Cas tools, optimized promoters, and glyco-engineering can further enhance product yield and homogeneity (Zhang et al., 2025; Kalkan et al., 2023; Xu et al., 2025; Huang et al., 2025; Zhang L. et al., 2024).

The following sections discuss the two major strategies employed in mammalian expression: stable production, the foundation of large-scale, long-term manufacturing and transient production, suited for rapid small-scale or research applications.

#### 2.1.1 Stable production of antibodies in mammalian cells

Stable integration of antibody genes into the host cell genome remains the defining feature of clinically-approved antibody manufacturing, with CHO cells serving as the most widely used platform for the production of therapeutic antibodies. While transient expression platforms offer clear advantages for rapid, small-scale production and early candidate screening, stable expression systems outperform them in scalability, long-term productivity, genetic uniformity, and batch-to-batch consistency. Thus, the choice between transient and stable expression largely depends on project objectives: transient approaches are ideal for fast, exploratory research, whereas stable cell lines are preferred for industrial-scale production and robustness. An overview of the antibodies produced in stable mammalian cell lines is shown in Table 1. Additionally an overview of clinically-approved antibodies produced in CHO cells is shown in Supplementary Table 1.

**TABLE 1:** Mammalian cell host, stable transfected, reported for Monoclonal Antibody (mAb) expression. N.d.: not disclosed.

Host	Antibody format	Antigen	Yield	Notes	References
CHO	IgG1	HER2	4.24 g/L	Fed-batch (trastuzumab)	Boulenouar et al. (2023)
CHO	IgG4	n.d.	8 g/L	Fed-batch	Weiß et al. (2025)
CHO	IgG1	TNFα	1 g/L	Fed-batch	Voronina et al. (2016)
CHO	n.d. (various monoclonal antibodies)	n.d.	4-∼9 g/L	Fed-batch	Gagnon et al. (2011)
CHO	n.d. (various monoclonal antibodies)	n.d.	4-∼9 g/L	Fed-batch	Lu et al. (2013)
CHO	IgG1	vascular endothelial growth factor (VEGF-A)	n.d.	Fed-batch	Grisanti and Ziemssen, (2007)
CHO	IgG1	CD20	n.d.	Fed-batch	Dorvignit et al. (2012)
CHO	IgG4	IL-4R	n.d.	Suspension culture	D’Ippolito and Pisano, (2018)
CHO	IgG1	n.d.	12 g/L	Fed-batch (CHO-MK cells)	Saeki et al. (2025)
CHO	n.d.	PD1	16.79 g/L	Perfusion culture method	Liang et al. (2023)
NS0	IgG1	complement C2	n.d.	Batch	Dharshanan et al. (2014)
NS0	n.d.	n.d.	2.64 g/L	Fed-batch	Burky et al. (2007)
PER.C6	IgG1	n.d.	8 g/L	Fed-Batch	Kuczewski et al. (2011)
PER.C6	IgG	n.d.	300–500 mg/L	Non fed-batch (in protein free medium)	Jones et al. (2003)
PER.C6	IgM	n.d.	0.5–2 g/L	Fed-batch	Tchoudakova et al. (2009)
SP2/O	IgG1	TNFα	2 g/L	Fed-batch	Beck, (2011)
SP2/O	IgG1	EGFR	750 mg/L	Fed-batch	Sauer et al. (2000)

A key consideration in establishing stable expression is the design of the expression vector, particularly the choice of promoters that drive heavy- and light-chain transcription. Common promoters are phosphoglycerate kinase 1 (PGK), human elongation factor-1 alpha (EF-1), and human ubiquitin C (UBC), as well as heterologous viral promoters like cytomegalovirus immediate early (CMV-IE) and Simian Virus 40 (SV40) ones (Dhara et al., 2018). More recently, the Hspa5 promoter has gained attention due to its linkage to ER stress–responsive pathways and its association with enhanced antibody productivity (Tanemura et al., 2022). Because Hspa5 activity is coordinated with the induction of ER chaperones and oxidative isomerases, its use can align antibody synthesis with cellular mechanisms that maintain protein-folding capacity, reduce ER stress–induced apoptosis, and sustain high-level production across passages and fed-batch phases.

Chinese hamster ovary (CHO) cells remain the dominant mammalian platform for monoclonal antibody (mAb) manufacturing due to their adaptability to serum-free suspension culture, capacity for human-like glycosylation, and well-established regulatory track record. Regulatory agencies are familiar with CHO-derived products, which simplifies comparability assessments and lifecycle management compared with alternative hosts (Yang et al., 2022). The importance of CHO cells in antibody production is outlined by the fact that in 2019, eight of the top ten drugs by global sales were biopharmaceuticals, seven of which were monoclonal antibodies produced in CHO cells (Bhutani et al., 2021). Moreover, existing data underscore the dominance of mammalian expression systems for mAb production, with nearly 89% of mammalian-derived products manufactured using CHO cells. This prevalence highlights the well-established advantages of the CHO platform, including its capacity to achieve high production titers of approximately 3–8 g/L at manufacturing scale (Walsh and Walsh, 2022).

Beyond their historical use, CHO cells offer broad genetic tractability, supporting iterative rounds of host cell engineering, targeted integration, and process-specific optimization. Contemporary CHO platforms incorporate defined parental lines, such as CHO-K1, CHO-S, and GS-knockout derivatives, which are pre-characterized for growth, metabolism, and product quality, thereby shortening development timelines (Srila et al., 2023). On the other hand, NS0, SP2/0, and PER.C6 cell lines are among the most efficient host systems apart from CHO cell line for mAb production (Dumont et al., 2016). Although SP2/0 cells exhibit expression levels approximately fivefold lower than CHO cells, their distinct glycosylation profiles can significantly influence mAb activity, immunogenicity, pharmacokinetics, and half-life, making them suitable for selected antibodies (Jaffaraghaei et al., 2024). However, non-human mammalian cell lines may introduce non-human post-translational modifications such as galactose-α1,3-galactose and N-glycolylneuraminic acid, which has led biosimilar developers to preferentially employ the same host cell line as the originator product, with SP2/0 cells currently used for mAbs including Abciximab, Basiliximab, Canakinumab, Cetuximab, Infliximab, Golimumab, and Ustekinumab (Reichert, 2012). Apart from traditional expression systems, a few studies have explored a human amniocyte-derived platform (CEVEC’s Amniocyte Production cell line (CAP®) and CAP-T® cells), to expand the repertoire of gene expression hosts by enabling rapid, flexible protein production with stable expression and without reliance on classical selection markers. Additionally, CAP-T® cells further enhance this system through SV40 large T antigen–mediated episomal replication, supporting high-efficiency transient expression while maintaining compatibility with CAP® cells for scalable stable production (Schiedner et al., 2008; Fischer et al., 2012).

One of the central considerations in stable cell line–based production is the careful choice of an appropriate selection system. Among the available strategies, dihydrofolate reductase (DHFR)– and glutamine synthetase (GS)–based selection systems continue to form the backbone of stable CHO cell line development (Zhang et al., 2022). These platforms are widely adopted due to their robustness, scalability, and proven track record in industrial biomanufacturing. Importantly, each system offers distinct advantages and trade-offs with respect to selection stringency, gene amplification behavior, and overall process complexity. As such, the selection system must be aligned with the specific expression goals, timeline, and manufacturing requirements of the production process. DHFR and GS-based selection systems continue to be central focus to stable cell line development in NS0 and other cell lines as well, apart from CHO cell lines. Advances in vector design, including bicistronic constructs and optimized selection workflows, have improved efficiency by enabling more reliable identification of high-producing clones. Two-stage selection strategies, supported by metabolic profiling and high-throughput screening, further enrich top producers and increase overall system yields (Toporova et al., 2023). Additionally, the emergence of site-specific integration technologies, such as recombinase-mediated cassette exchange, now allows precise genomic targeting, improving expression consistency and product quality (Szkodny and Lee, 2025). These developments reinforce the concept that while integration of locus, host-cell background, and transgene architecture define baseline expression, regulatory elements can create localized transcriptional environments that further enhance performance.

Recent studies have also shown that full-gene deletion of GS using CRISPR/Cpf1 can produce CHO hosts with markedly improved selection stringency and antibody productivity compared with wild-type cells. In CHO-S, for example, single-gene GS5 knockout was sufficient to obtain bulk pools with significantly higher mAb titers, whereas in CHO-K1 compensatory upregulation of a second GS isoform necessitated more extensive engineering to maintain selection efficiency (Srila et al., 2023). Comprehensive characterization of GS-knockout CHO (GS-KO) lines indicates that productivity gains are not solely explained by gene copy number, but also by enhanced transcriptional activity and favorable shifts in cellular metabolism under glutamine-free, high-stringency conditions. Engineered promoter variants, such as weakened SV40E derivatives adapted to GS-KO hosts, can further increase the efficiency of generating high-producing clones, reducing screening burden and improving the overall robustness of GS-based platforms (Fan et al., 2013).

Collectively, these innovations have elevated productivity, reproducibility, and scalability, solidifying stable cell line-based expression as the industrial standard for therapeutic antibody manufacturing. In recent examples, moving beyond traditional methods for determining growth conditions, the application of machine learning to optimize them has produced notably improved outcomes (approximately 48% higher yield than the existing approach) (Richter et al., 2025). By analyzing historical and conventional datasets and constructing artificial neural networks through machine learning (ML) techniques, researchers have achieved substantial enhancements that may hold the key to resolving various longstanding challenges.

Apart from upstream improvements, i.e., developments to improve cell line selection procedures, increased mAb productivity and quality and transition from batch productions to continuous productions enabled by perfusion bioreactors, further enhancements can be attained improving downstream processing, i.e., all the purification steps necessary to obtain a final drug product (Khanal and Lenhoff, 2021). Traditionally after large scale fermentation, downstream processing of the production batch involved clarification, purification, polishing, viral clearance and final filtration and concentration steps (Saha et al., 2025). All these steps are intended to remove any possible impurities from the manufactured mAb that originate from the cell line used for production, such as host cell proteins and DNA, as well as other impurities such as endotoxins, viruses, high-molecular-weight aggregates, and other smaller impurities. Since this topic is very broad and goes beyond the scope of this review, only a few example improvements are discussed here. For a more complete overview, readers are referred to other detailed reviews and opinion papers on the subject (Kelley, 2024; Kelley et al., 2021; Carvalho et al., 2026; Matte, 2022; Zarzar et al., 2023; Khanal and Lenhoff, 2021). As mentioned above, after the cultivation step, the producing cells need to be separated from the secreted mAb. Traditionally this has been achieved through centrifugation (namely disk stack centrifuges) and filtration before Protein A purification. However, cells subjected to high shear forces rupture and release all their cellular content (majorly DNA and host cell proteins), which, in turn, contaminate the preparation. Furthermore, these debris can also easily clog filters in the filtration step before affinity chromatography. Therefore, approaches like precipitation and flocculation have been investigated, to allow easier removal of these impurities (Saballus and Kampmann, 2022). Indeed, flocculation in the investigated settings produced the best results in terms of reduction of host cell DNA contamination and better filterability and higher filter duration (Saballus and Kampmann, 2022).

Additionally, integrated continuous manufacturing and hybrid bioprocesses can also serve as additional examples of potential improvements in downstream processing (Coffman et al., 2021; Narayanan et al., 2022; Reger et al., 2023). Indeed continuous counter-current chromatography has allowed maximal resin utilization, without exceeding its breakthrough capacity, decreased timing and with better economics than classical stepwise purifications (Shi et al., 2021; Angarita et al., 2015). To the best of our knowledge this kind of set-up has been tested in industrial pilot settings but not yet transitioned to full scale industrial production (Xu et al., 2020).

#### 2.1.2 Transient production of antibodies in mammalian cells

In addition to the production of recombinant antibodies with a focus on high yields through stable mammalian cell lines, preclinical testing and non-therapeutic applications often require the rapid and parallel production of a large number of different antibody clones. Transient expression of recombinant antibodies in mammalian cell lines is an established method that enables rapid and efficient production of functional antibodies (Geisse and Fux, 2009). An overview of antibodies produced in mammalian cells by transient gene expression (TGE) is shown in Table 2.

**TABLE 2:** Mammalian cell host, transient transfected, reported for Monoclonal Antibody (mAb) expression. N.d.: not disclosed.

Host	Antibody format	Antigen	Yield	Notes	References
HEK293-E6	scFv-Fc	n.d.	600 mg/L	Optimized expression vectors and fed-batch	Jäger et al. (2013)
EXPI293F	scFv-Fc	Diphtheria Toxin	n.d.	PEI, Erlenmeyer flasks	Wenzel et al. (2020)
	IgG		n.d.	PEI, Erlenmeyer flasks	
EXPI293F	scFv-Fc	SARS-CoV-2 Spike	20–440 mg/L	PEI, flat-bottom tubes	Bertoglio et al. (2021b)
	IgG		12–93 mg/L	PEI, Erlenmeyer flasks	Bertoglio et al. (2021a)
HEK293E	IgG	PD1	90 mg/L	PEI, Erlenmeyer flasks	Ding et al. (2017)
HEK293E	IgG	RhD	>1 g/L	PEI, square-shaped glass bottles	Backliwal et al. (2008)
CHO-K1	IgG1	n.d.	n.d.	Amaxa Nucleofector System, 24 shallow-well plates	O’Neill et al. (2023)
CHO-S	Adalimumab	TNF-alpha	18mg/L	Lip2000 Transfection Reagent	Yang et al. (2022)
Expi293-F	hIgG	PD-L1	353 mg/L	Expifectamine	Carrara et al. (2021)
Expi293F	IgM	V3/gp120	200 mg/L	ExpiFectamine	Gong et al. (2022)
HEK293F	cetuximab	EGFR	57 mg/L	Novel cationic tetraamine lipid (spermine lipid) as transfection reagent	Sui et al. (2023)

In most cases, the genes for the antibody heavy and light chains are introduced into CHO or HEK293 cells using suitable expression vectors and transfection reagents such as calcium phosphate (Meissner et al., 2001), cationic lipids or PEI (Thomas and Klibanov 2002). Physical transfection methods, such as biolistic protocols using gold particles, play only a minor role in cell transfection for antibody production, as they require a sophisticated instrumental setup. Those are more commonly used for transgene expression in epithelial cells (Li et al., 2013).

Stable integration into the genome is not necessary; the transfected DNA remains episomal in the cell nucleus and is transcribed there for a limited period of time, allowing the desired recombinant protein to be produced quickly. Protein synthesis typically occurs within a few days after transfection. TGE conditions have to be evaluated carefully due to significant influence of cell density, PEI concentration and the ratio of light and heavy chain encoding plasmids (Bollin et al., 2011).

Biotechnical production of antibodies ranges from small scale in microtiter plates to hundreds of liters batch or fed-batch processes with non-adherent cell growth (Baldi et al., 2007) leading to expression yields of up to 600 mg/L for scFv-Fc in HEK293 cells (Jäger et al., 2013).

Various opportunities for optimization and adaptation have also opened up at the DNA level in recent years: vector systems with strong promoters (e.g., CMV promoter) are frequently used; elements such as Epstein-Barr virus nuclear antigen 1 (EBNA1) are used to further increase expression (Loignon et al., 2008; Zhang et al., 2009).

Nowadays, artificial neural networks (ANNs) and genetic algorithms are increasingly used to predict and optimize recombinant protein yields of antibody fragments under different culture medium conditions, offering an alternative to traditional response surface methodology (RSM). In a recent study, the ANN model (259.83 mg/L) aligned more closely with experimental data (259.51 mg/L) than the RSM approach (276.13 mg/L) (Basafa et al., 2024).

Machine learning-supported DNA sequence engineering and the selection of genes that encode protein-specific signal peptides are also regularly used in mammalian vector engineering to increase the yield of secretory proteins, as demonstrated with 37 peptides of different origins: for all products tested it was possible to derive vector designs that enhanced product titers by > 1.8-fold, compared to standard industry technologies (O’Neill et al., 2023).

### 2.2 Yeasts

Eukaryotic cells possess advanced machinery for protein folding, post-translational modification, and secretion, which enables efficient production of antibodies, including full-length immunoglobulins, compared to bacteria. Among eukaryotic microorganisms, yeasts combine these cellular advantages with short generation times, ease of genetic manipulation, and robust growth on simple media. Their long history of use in food fermentation, absence of bacterial endotoxins, and “Generally Recognized as Safe” (GRAS) status further support their application in therapeutic protein production (De Pourcq et al., 2012; Schreuder et al., 1996). An overview of the antibodies produced in yeasts is shown in Table 3. Among yeast species, *K. phaffii* has emerged as the most widely used host for the production of pharmaceutical proteins, including different antibody formats. Other yeasts, such as *S. cerevisiae*, *Hansenula polymorpha*, *Yarrowia lipolytica*, *Arxula adeninivorans*, *Kluyveromyces lactis*, and *Schizosaccharomyces pombe*, have been employed for recombinant protein production, but their role in antibody expression remains limited (Baghban et al., 2019; Mattanovich et al., 2012). *K. phaffii* offers several practical advantages for antibody production, e.g. it can reach very high cell densities exceeding 100 g/L dry weight under respiratory growth, while maintaining low contamination from endogenous proteins, which simplifies downstream processing (Gasser et al., 2006).

**TABLE 3:** Yeast strains reported for Monoclonal Antibody (mAb) expression.

Host	Antibody format	Antigen	Yield	Notes	References
*Pichia pastoris (now Komagataella phaffii)*	scFv-based N-terminal trimerbody	Human carcinoembryonic antigen (CEA)	6 mg/L	500 mL in 2,500 mL baffled flasks	Blanco-Toribio et al. (2014)
*K. phaffii*	IgG	HER2	0.5 g/L	Transglycosilation post production	Liu et al. (2018)
*K. phaffii*	IgG1	HIV-VRC1	3.05 mg/L	50 mL in 500 mL baffled flasks	Aw et al. (2017)
*K. phaffii*	scFv	CD22	25 mg/L	Shaking flasks	Zarei et al. (2014)
*S. cerevisae*	VHH, scFv (pexelizumab), Fab (ranibizumab)	Lysozyme, Complement C5, VEGF-A	Low yield (Medium had to be concentrated to detect mAbs)	​	Wang et al. (2021)
*K. phaffii*	scFvs (LR, 10FG2)	*C. noxius/C. sculpturatus* toxin (Cn2, CsEM1a)	20–44 mg/L	500 mL in 2,500 mL shaking flasks	Gómez-Ramírez et al. (2023)
*K. phaffii*	Single-chain diabody (scDb)	hERG1, β1-Integrin	1.6–4.1 mg	​	Duranti et al. (2021)
*K. phaffii*	scFv	Amyloid-β	20.5 mg/L	​	Montoliu-Gaya et al. (2017)
*K. phaffii*	scFv	PSMA	165 mg/L	2L Fermenter	Parker et al. (2013)

Ridder et al. demonstrated the production of scFvs in yeast (Ridder et al., 1995). Yields for various scFvs ranged from 250 to 300 mg/L (Khatri et al., 2011; Eldin et al., 1997). Under optimized bioreactor conditions and with co-expression of the chaperone immunoglobulin-binding protein (BiP), functional scFv production could reach up to 8 g/L (Damasceno et al., 2007).

Llama-derived VHHs expressed in *S. cerevisiae* achieved yields exceeding 100 mg/L in shake flask cultures (Frenken et al., 2000). More complex but still single-gene-encoded formats, such as dimeric scFv-Fc antibodies, reached production levels of 60–70 mg/L in *P. pastoris* shake cultures (Wan et al., 2013).

Antibody formats requiring two genes, such as Fab and full-length IgG, necessitate the fusion of each chain with an N-terminal secretory signal sequence and co-transformation of both constructs. Limited data are available on full-length IgG expression in yeast. Early studies reported the production of a mouse-human chimeric antibody and its Fab fragment in *S. cerevisiae*, with yields of 50–80 μg/L for IgG and 200 μg/L for Fab. The chimeric IgG mediated tumor-specific binding and antibody-dependent cellular cytotoxicity (ADCC), but not complement-dependent cytotoxicity (CDC) (Horwitz et al., 1988). Recent improvements in cell culture optimization enabled the production of 358 μg/L of full-length tocilizumab and 428 mg/L of its Fab fragment in *K. phaffii* shake cultures (Das et al., 2025). In large-scale bioreactors (1,200 L), yields of up to 1.6 g/L of human IgG1 were achieved (Ye et al., 2011).

Challenges associated with heterologous protein expression in yeast can be mitigated by codon optimization, promoter and signal peptide engineering, overexpression of chaperones, or glyco-engineering (Juturu and Wu, 2018). Production of antibody fragments has also been enhanced through DNA shuffling (Linden et al., 2000; Cross et al., 2023). Heterologous gene expression can impose a substantial metabolic burden. This can lead to altered metabolic profiles, reduced respiratory capacity, slower growth, lower yields, and misfolding of the expressed antibodies (Glick, 1995; Seresht et al., 2013; Ruijter et al., 2018). In yeast, proper folding becomes a major bottleneck when recombinant antibodies are expressed at high levels (Zahrl et al., 2019). Inefficient secretion of large heterologous proteins (>30 kDa), proteolysis of secreted proteins during high-cell-density fermentation, and inappropriate glycosylation of human glycoproteins present additional challenges, prompting extensive engineering of yeast strains.

Overexpression of the chaperones BiP or PDI in *S. cerevisiae* has been shown to increase scFv secretion 2 to 8-fold, achieving average titers of 20 mg/L in shake-flask cultures (Shusta et al., 1998). Similarly in *Ogataea minuta*, co-overexpression of protein disulfide isomerase (OmPDI1), thiol oxidase (OmERO1), and the KAR2 protein (OmKAR2) enhanced fully assembled H2L2 (IgG) antibody titers by a factor of 16, reaching 30 mg/L compared with the parental strain (Suzuki et al., 2017).

ER engineering also provides opportunities to improve antibody production. In *S. cerevisiae*, deletion of the lipid-regulatory gene *OPI1* expanded the ER and increased IgG secretion 4-fold relative to the wild type. When combined with overexpression of the peptidyl-prolyl isomerase CPR5, IgG production improved 10-fold compared to wild-type levels (Ruijter et al., 2016).

Different glycosylation patterns in yeasts compared to humans also constitute a hurdle for mAb production and further development. Hypermannosylated N-glycans are highly immunogenic in humans and are accompanied by low fucosylation and an absence of terminal sialic acids (Ghaderi et al., 2012). *K. phaffii* naturally exhibits lower levels of hyperglycosylation than *S. cerevisiae*, and its N-linked glycans resemble the mammalian high-mannose core Mannose5–6N-acetylglucoseammine6 (Grinna and Tschopp, 1989). To address these limitations, glyco-engineered *K. phaffii* strains capable of producing humanized glycosylation patterns have been developed (Jacobs et al., 2009; Hamilton et al., 2006; Shenoy et al., 2021). Therapeutic IgG antibodies produced in these strains show properties comparable to trastuzumab generated in mammalian cells (Zhang et al., 2011). Unlike IgGs from wild-type yeast, antibodies produced in glyco-engineered strains can trigger antibody-mediated effector functions. Efforts to optimize production processes using such strains are underway for both commercial antibody manufacturing (Ye et al., 2011) and high-throughput screening applications (Jiang et al., 2010). In addition to cellular engineering, enzymatic approaches offer complementary options. *In vitro* deglycosylation of trastuzumab, produced in *K. phaffii*, followed by targeted transglycosylation of human N-glycans using endoglycosidases represents an alternative strategy to obtain defined glycan structures (Liu et al., 2018). These efforts culminated in February 2020, when *K. phaffii*- produced Eptinezumab, a humanized, rabbit-derived, aglycosylated IgG1 that inhibits the human calcitonin gene-regulated peptide (CGRP), was approved for the prevention of migraine (Dhillon, 2020; Garcia-Martinez et al., 2020). Nevertheless, commercially approved antibodies expressed in yeast remain an exception.

### 2.3 Filamentous fungi

Filamentous fungi such as *Aspergillus* and *Trichoderma* have strong secretion capabilities. Accordingly, they are attractive industrial host systems for the production of endogenous as well as heterologous proteins and metabolites (Ward, 2012; Bischof et al., 2016). Today, the use of filamentous fungi for the production of compounds such as citric acid and cellulases is already well established (Mores et al., 2021; Bischof et al., 2016). Both citric acid produced by *Aspergillus niger* and cellulase produced by *Trichoderma reesei* are listed as GRAS under Title 21 of the Code of Federal Regulations (United States), but GRAS notices are also filed for several other products of filamentous fungi.^1^

The production of antibodies as well as many other heterologous proteins has been primarily achieved using the endogenous promoters of glucoamylase A (GlaA), 1,4-β-endoxylanase A (ExlA) (in *Aspergillus* spp. (Ward et al., 2004; Joosten et al., 2005b; Joosten et al., 2005a; Sotiriadis et al., 2001; Frenken et al., 1998)) or cellobiohydrolase I (CBHI) (in *T. reesei* (Nyyssönen et al., 1993; Arai et al., 2023)). In addition to the signal sequences of endogenously secreted proteins, a secreted carrier protein is often used to improve antibody production. Full length or truncated versions of GlaA (Frenken et al., 1998; Sotiriadis et al., 2001; Ward et al., 2004), CBHI/II (Nyyssönen et al., 1993; Arai et al., 2023) or α-amylase (AmyB) (Huynh et al., 2020) are mostly fused to the N-terminus of the protein of interest. Importantly, fusion of the antibody chain(s) to a carrier protein does not necessarily mean that a fusion protein is secreted by the fungus: the introduction of a Kexin-protease cleavage site in the linker between the carrier protein and the antibody leads to cleavage of the protein of interest from the carrier protein prior to secretion. However, cleavage efficiency was dependent on the cultivation conditions and cleavage site. This resulted in the secretion of antibody-carrier fusion proteins as well as antibodies (Ward et al., 2004; Landowski et al., 2016).

The previously described fungal expression systems are dependent on the endoplasmic reticulum (ER)-Golgi pathway. But it was also tried to harness the unconventional, i.e., ER-Golgi-independent, secretion of chitinase Cts1 in *Ustilago maydis* for the production of antibody fragments in the yeast form of the organism (Stock et al., 2012; Sarkari et al., 2014; Terfrüchte et al., 2017; Philipp et al., 2023). This circumvents any ER/Golgi-mediated post translational modifications and thus would also circumvent fungi-specific glycosylations. However, these attempts have thus far not been competitive with the highest reported yield of a VHH-Cts1 fusion protein being 140 μg/L (Terfrüchte et al., 2017).

One of the major challenges when using filamentous fungi for the expression of heterologous proteins is the degradation of those proteins by endogenously produced proteases. Accordingly, the addition of protease inhibitors to the culture medium can increase the yield of the produced proteins (Arai et al., 2023; Landowski et al., 2016). Furthermore, there have been efforts to reduce protease activity and thus improve the production yield by using protease-deficient strains (Yoon et al., 2011; Huynh et al., 2020; Havlik et al., 2017; Kaiser et al., 2024; Landowski et al., 2015; Landowski et al., 2016) or by adjusting cultivation conditions (Havlik et al., 2017). A combination of the use of a protease-gene deletion strain, of protease inhibitors and optimization of cultivation conditions could increase the production yield of Interferon alpha-2b to 4.5 g/L (Landowski et al., 2016). Still, the improvement of yields after addition of protease inhibitors highlights the need for further strain development.

Filamentous fungi perform N- and O-glycosylation of proteins but their high-mannose glycans differ from mammalian glycosylations which can impact the activity of glycoproteins like antibodies (Nevalainen and Peterson, 2014). For several antibodies produced in *A. niger, A. oryzae* and *Thermothelomyces heterothallica* C1, mainly oligo-/high-mannose N-glycans were observed (Ward et al., 2004; Huynh et al., 2020; Kaiser et al., 2024). The deletion of the α-1,6-mannosyltranferase (Och1) in *A. oryzae* led to a reduced molecular weight of the N-glycans in comparison to the parental strain (Huynh et al., 2020). However, glyco-engineering to “humanize” the glycosylation pattern of antibodies produced in filamentous fungi has not yet been performed. In general there are few reported attempts of the humanization of N-glycosylation in fungi (Kainz et al., 2008; Natunen et al., 2017; Anyaogu et al., 2021). An anti-SARS-CoV-2 antibody HuMab 87G7 (Du et al., 2022) produced in *T. heterothallica* exhibited similar antigen binding and SARS-CoV-2 neutralization but increased NK-cell activation as the same antibody produced in mammalian cells. The latter was hypothesized to be from a lack of fucosylation of the fungi-produced antibody. It also conferred prophylactic protection against SARS-CoV-2 *in vivo* (Kaiser et al., 2024). However, the impact of glycosylation differences between fungal and mammalian production systems requires further investigation.

The first antibody fragment that was produced in filamentous fungi was a Fab fragment fused to CBHI as a carrier protein produced in *T. reesei*. The yield of the fusion protein was 150 mg/L in a 15 L bioreactor (Nyyssönen et al., 1993). Since then, several VHH and scFv antibody fragments have been produced yielding up to 508 mg/L of a bivalent anti-von Willebrand factor VHH (caplacizumab) in *T. reesei* (Arai et al., 2023) and reportedly >200 mg/L of an anti-*Streptococcus oralis* scFv in *A. awamori* (now *A. luchuensis*) (Frenken et al., 1998). Moreover, humanized and human IgGs like trastuzumab and adalimumab were produced in *Aspergillus* spp. and showed similar binding to their targets as the commercial antibodies (Ward et al., 2004; Huynh et al., 2020). The best production yield of a full antibody produced in filamentous fungi so far is that of HuMab 87D7 with 1.6 g/L produced in *T. heterothallica* in a 1 L bioreactor (Kaiser et al., 2024). While this seems promising, filamentous fungus-based expression systems need to be further improved to be real competitors to mammalian cells in antibody production. An overview of the antibodies produced in filamentous fungi is shown in Table 4.

**TABLE 4:** Filamentous fungi host reported for Monoclonal Antibody (mAb) expression.

Host	Antibody format	Antigen	Yield	Notes	References
*Aspergillus awamori* (current name *A. luchuensis*)	VHH	Azo-dye Reactive Red 6 hapten (RR6)	≤7.5 mg/L	Addition of BSA to decrease protease activity	Joosten et al. (2005a)
*A. luchuensis*	VHH (R9)	Azo-dye Reactive Red 6 hapten (RR6)	≤30 mg/L (ARP-R9)	*Arthromyces ramosus* peroxidase (ARP) fused to C-terminus of VHH	Joosten et al. (2005b)
*A. luchuensis*	scFv (D1.3)	Lysozyme	≤108.9 mg/L	GlaA fusion, cleavage site, 7 L fermenter	Sotiriadis et al. (2001)
*A. luchuensis*	scFv (D1.3), scFv (4715)	Lysozyme, SSP5 surface protein from *S. oralis*	>200 mg/L (4715)	Presence of multimers, GlaA/ExlA fusion, cleavage site, 10 L fermenter	Frenken et al. (1998)
*A. niger* var*. awamori*	Humanized IgG1 (trastuzumab, Hu1D10), Fab’ (trastuzumab)	HER2 HLA-DR	≤900 mg/L (Trastuzumab), 1,200 mg/L (Trastuzumab Fab’)	GlaA fusion, cleavage site	Ward et al. (2004)
*A. oryzae*	VHH	EGFR	73.8 mg/L	N-terminal region from *Rhizopus oryzae* lipase fused to VHH	Okazaki et al. (2012)
*A. oryzae*	IgG (anti-CD20, trastuzumab)	CD20, HER2	1.3 mg/L (anti-CD20)	Wild type strains were used, GlaA fusion, cleavage site	Jiang et al. (2019)
*A. oryzae*	Human IgG1 (adalimumab)	TNFα	≤39.7 mg/L	10-protease-deletion strain, AmyB fusion, cleavage site	Huynh et al. (2020)
*Neurospora crassa*	scFv (HT186-D11)	MUC1	≤3 mg/L (Fusion protein) calculated to be 0.9 mg/L of scFv	4-fold protease gene deletion, GlaA fusion, 10 L fermenter	Havlik et al. (2017)
*Thermothelomyces heterothallica* (C1)	Human IgG1 (HuMab 87G7)	RBD SARS-CoV-2 S-protein	≤1,600 mg/L	14 protease-gene deletion strain, 1 L fermenter	Kaiser et al. (2024)
*Trichoderma reesei*	Fab	2-phenyloxazolone	≤150 mg/L of CBH1-Fab	CBHI fusion, only linked to heavy chain, 15 L fermenter	Nyyssönen et al. (1993)
*T. reesei*	IgG (anti-CD20)	CD20	2 mg/L anti-CD20	Wild type strains were used, CBH1 fusion, cleavage site	Jiang et al. (2019)
*T. reesei*	VHH (1ZVH (monovalent), caplacizumab (bivalent), ozoralizumab (trivalent, bispecific))	Lysozyme (1ZVH), vWF (caplacizumab), TNFα/HAS (ozoralizumab)	≤508 mg/L (caplacizumab)	Inducer-free strain, CBHII fusion, cleavage site	Arai et al. (2023)
*Ustilago maydis* (in yeast form)	VHH	GFP Botulinum toxin A	0.14 mg/L αBoNTANB-Cts1	*kex2* deletion strain, Cts1 fusion, *Tobacco Etch Virus* protease cleavage site	Terfrüchte et al. (2017)

### 2.4 Microalgae as hosts for antibody production

The use of microalgae as hosts for protein expression was firstly demonstrated over two decades ago (Sun et al., 2003). Since then, these organisms have attracted increasing attention as versatile, resilient, and potentially cost-effective platforms for biopharmaceutical production. Significant advances have established microalgae as promising systems for the expression of complex proteins, including monoclonal antibodies. Most antibody products reported to date from microalgal platforms are full-length IgG molecules, although derivatives such as scFv-Fc have also been demonstrated (Mayfield et al., 2003; Mayfield and Franklin, 2005; Tran and Mayfield, 2010). While microalgae can assemble and in some species such as *Phaeodactylum tricornutum* secrete fully folded human IgG, their N-glycosylation patterns differ from those of mammalian expression systems. Algal hosts typically produce oligomannosidic or paucimannosidic N-glycans, often containing plant-type β1,2-xylose and α1,3-fucose residues and lacking terminal galactose/sialic acid (Dumontier et al., 2018; Barolo et al., 2020). Ongoing glyco-engineering efforts aim to “humanize” these pathways to achieve more comparable glycoforms to mammalian cells like CHO (Barolo et al., 2020). Several species, such as *Chlamydomonas reinhardtii, Chlorella vulgaris, Dunaliella salina* and *Haematococcus pluvialis,* are particularly attractive owing to their GRAS status and their long-standing use in food, cosmetic, and pharmaceutical industries (Hempel et al., 2011; Demurtas et al., 2013; Dyo and Saul, 2018). Since the initial use of microalgae as a production system, a variety of reviews and book chapters have discussed and evaluated their application and potential in industrial production and biotechnology (Yıldırım, 2023; Rajput et al., 2024; Muñoz-Solórzano et al., 2024; Trujillo et al., 2024). Among the microalgal genera, representatives of the *Chlorophyta* and *Bacillariophyta* have emerged as the most extensively explored hosts for recombinant protein production (Gangl et al., 2015; Diaz-Garza et al., 2024; Rajput et al., 2024). An overview of the antibodies produced in microalgae is shown in Table 5.

**TABLE 5:** Microalgae hosts reported for Monoclonal Antibody (mAb) Expression. N.d.: not disclosed.

Host	Antibody format	Antigen	Yield	Notes	References
*C. reinhardtii*	scFv-Fc	HSV glycoprotein D	n.d.	First antibody expressed in plastid, fully active	Mayfield et al. (2003)
*C. reinhardtii*	IgG	Anthrax PA83	n.d.	Assembly and binding confirmed	Tran et al. (2009)
*P. tricornutum*	IgG	HBV surface antigen (HBsAg)	0.45–2.5 mg/L	First demonstration of secreted IgG	Hempel and Maier, (2012)
*P. tricornutum*	IgG	Marburg virus NP	0.5–1.3 mg/L	Codon optimization improved titers	Hempel et al. (2017)

To enable recombinant antibody production, several transformation methods have been developed, however, the method of choice depends on the targeted organelle. For instance, nuclear, chloroplast or mitochondrial transformation can be used (Yıldırım, 2023; Muñoz-Solórzano et al., 2024). Nuclear transformation enables the production of antibodies with post-translational modifications such as N-glycosylation (Barolo et al., 2020; Yıldırım, 2023; Rajput et al., 2024), whereas chloroplast transformation yields aglycosylated antibodies (Yıldırım, 2023; Rajput et al., 2024), which can be advantageous for therapeutic applications where Fc-mediated effector functions, such as ADCC, are undesired or be used as diagnostic tools, where Fc-mediated effects are not needed. Although mitochondrial transformation has been demonstrated (Larosa and Remacle, 2013), it has not yet been applied to the production of recombinant proteins or antibodies and remains an emerging experimental technology. Microalgal transformation can be achieved through a range of different methods, including DNA-coated nanoparticles, CRISPR/Cas9 editing, electroporation, biolistic bombardment, *Agrobacterium*-mediated transfer, *E.coli*-mediated transfer, glass bead agitation, natural transformation, liposome delivery and sonoporation (Kindle, 1990; Chen and Lee, 2019; Naduthodi et al., 2021; Sreenikethanam et al., 2022; Liu et al., 2022; Muñoz-Solórzano et al., 2024; Rajput et al., 2024). Biolistic bombardment remains the gold standard for chloroplast engineering in *Chlorophyta* (Kindle, 1990; Mayfield et al., 2003), while nuclear transformation is typically performed by electroporation or glass-bead agitation in *C. reinhardtii* (Kindle, 1990; Naduthodi et al., 2021) and by *Agrobacterium* or PEG-mediated delivery in other green algae (Trujillo et al., 2024). In *Bacillariophyta*, bacterial conjugation now surpasses electroporation for stable nuclear expression and is combined with episomal vectors, CRISPR/Cas9, and synthetic regulatory elements to improve expression stability and overcome silencing (Naduthodi et al., 2021; Diaz-Garza et al., 2024; Trujillo et al., 2024; Rajput et al., 2024). Although the use of microalgae for monoclonal antibody (mAb) production is still limited, several companies and start-ups have emerged that specialize in developing these systems. For example, Phaeosynt GmbH (Germany) and Alga Biologics (France) both are developing *P. tricornutum* based systems for antibody production. In addition, a 2013 patent filed by Philipps-Universität Marburg (EP2671950A1) demonstrated that microalgae such as *P. tricornutum* can be engineered to secrete fully assembled and functional proteins efficiently and directly into the culture medium. The patent has since been withdrawn, allowing the described approach to be used publicly. Microalgae may complement or, in specific cases, replace traditional hosts when cost, sustainability, or unique product properties are decisive. With the growing interest in “vegan biologics”, particularly in diagnostics and the emergence of commercial players, microalgae are increasingly positioned as competitive platforms for animal-free antibody production.

#### 2.4.1 Antibody expression in *Chlorophyta* (green algae)

The model chlorophyte *C. reinhardtii* has been the principal representative of this group in recombinant antibody production. Antibody genes can be stably integrated into either the nuclear or the chloroplast genome, allowing good expression (Mayfield et al., 2003; Mayfield and Franklin, 2005; Hempel et al., 2011). Using the chloroplast strategies, a variety of antibody formats have been successfully expressed, including scFv-Fc fusion proteins (Mayfield et al., 2003), full-length human IgG1 (Tran et al., 2009) and fusion proteins (Franklin et al., 2002; Mayfield and Schultz, 2004). Beyond *C. reinhardtii,* other chlorophyta such as *C. vulgaris, D. salina,* and *H. pluvialis* are of interest due to their GRAS status and long history in food and nutraceutical applications (Dyo and Saul, 2018; Rajput et al., 2024). Although antibody expression has not yet been demonstrated in these species, they have been successfully transformed for production of other recombinant proteins and metabolites (Yıldırım, 2023; Trujillo et al., 2024; Muñoz-Solórzano et al., 2024), suggesting potential as future antibody hosts. Ongoing advances in genome editing and synthetic biology are enhancing the genetic tractability of chlorophytes, with recent applications of CRISPR-Cas9 and modular promoter libraries in *C. reinhardtii* overcoming transgene silencing and improving expression stability (Naduthodi et al., 2021). In parallel, efforts in glyco-engineering aim to establish more mammalian-like glycosylation patterns (Dumontier et al., 2018; Barolo et al., 2020). These developments underline the broader potential of chlorophytes as sustainable antibody production platforms.

#### 2.4.2 Antibody expression in *Bacillariophyta* (diatoms)

Diatoms represent a second group of microalgae with rapidly growing biotechnological relevance. Among these, *P. tricornutum* has become the best-studied species. Hempel et al. (2011) demonstrated the expression of a full-length human monoclonal antibody in *P. tricornutum,* establishing this diatom as a viable host system (Hempel et al., 2011). Remarkably, *P. tricornutum* is capable of secreting fully assembled human IgG into the culture medium, achieving yields of up to ∼2.5 mg/L after 5 days cultivation (Hempel and Maier, 2012). However, more improvements are required to reach industry-competitive yields. Beyond *P. tricornutum,* several other diatoms have been engineered for recombinant protein production, including *Cyclotella cryptica, Navicula saprophila, Cylindrotheca fusiformis,*and *Thalassiosira* species (Dunahay et al., 1995; Fischer et al., 1999; Rajput et al., 2024). Diatoms, especially certain morphotypes of *P. tricornutum*, appear to exhibit relatively strong capacities for protein secretion (Galas et al., 2021), a trait that may offer advantages relative to many chlorophytes, though a direct comparative quantification was not yet firmly established. With continued progress in strain engineering and bioprocess optimization, diatoms could provide a scalable, secretion-competent alternative to more established host systems for monoclonal antibody production. An overview of the antibodies produced in microalgae is shown in Table 5.

### 2.5 Protozoa

The flagellated protozoan *Leishmania* (subgenus *Sauroleishmania*) *tarentolae* is a parasite isolated from the Moorish gecko *Tarentola mauritanica*. It is an interesting alternative expression system, because it allows a mammalian-like glycosylation pattern. It includes O-glycosylation as well as N-glycosylation which is highly conserved in mammals, but does not include terminal sialic acids. Stable cell lines can be made by electroporation and the cells can be cultivated in shake flasks (Klatt et al., 2013; Lai et al., 2019; Zimna and Krol, 2024). This protozoan is classified as a BSL1 organism, but the strain LEM-125 can infect mammals transiently, therefore the strain TARII/UC should be preferred (Klatt et al., 2019). Leishmania was used for the production of different scFvs with a median production rate of up to 3.83 mg/L (max ∼6 mg/L) (Klatt and Zoltán, 2012) and scFvs fused to a rabbit Fc part resulting in up to 2.95 mg/L (Jørgensen et al., 2014). The production of a complete anti-CD20 IgG was successful with 4 mg/L (Jiang et al., 2019). A commercial Leishmania production system (LEXSY) is available by JenaBioscience. An overview of the antibodies produced in protozoa is shown in Table 6.

**TABLE 6:** Protozoa production of recombinant antibodies. N.d.: not disclosed.

Host	Antibody format	Antigen	Yield	Notes	References
*Leishmania tarentolae*	scFv	n.d.	0.04–3.83 mg/L	Expression of four different scFvs	Klatt and Zoltán, (2012)
*L. tarentolae*	scFv	C5a anaphylatoxin, human vimentin, murine laminin	0.3–1 mg/L	Expression of three different scFvs	Jørgensen et al. (2014)
*L. tarentolae*	scFv-Fc	human vimentin, murine laminin	0.6–2.95 mg/L	Expression of scFv rabbit Fc fusion proteins	Jørgensen et al. (2014)
*L. tarentolae*	IgG	CD20	4 mg/L	Expression of codon optimized rituximab	Jiang et al. (2019)

### 2.6 Insect cells

Insect cell expression systems are a vital platform for producing animal-free recombinant proteins, including antibodies. The system’s inherent eukaryotic advantages include correct folding, disulfide bond formation, and antibody assembly (Hasemann and Capra, 1990; Nesbit et al., 1992; Roldão et al., 2014). The most commonly used insect cell lines are Sf21 and Sf9, which are derived from *Spodoptera frugiperda*, and HighFive™ (Hi5, BTI-TN-5B1-4), which is derived from *Trichoplusia ni* (Vaughn et al., 1977; Granados et al., 1994; Fabre et al., 2020). These cell lines are well suited for large-scale applications due to their capacity for growth in low-cost (compared to mammalian cell production systems), serum-free suspension cultures at temperatures ranging from 25 °C to 28 °C without the need for CO_2_ supplementation. This enables the scaling up from laboratory sized cultures to commercial bioreactors ranging from 2 to 2,500 L. Sf21 and Sf9 cells are often used for generating high titer baculovirus stocks. In contrast, Hi5 cells are more frequently used to produce recombinant proteins due to their ability to achieve higher yields, while generating less infectious baculovirus (Davis et al., 1993; Kwon et al., 2003; Buckland et al., 2014; Wilde et al., 2014; Hong et al., 2022). Two primary approaches have gained significant traction in the field: the well-established Baculovirus Expression Vector System (BEVS) and the increasingly popular TGE method. BEVS has been demonstrated to be a robust, high-yield, and scalable system that can be applied to cultured insect cells or directly to insect larvae and pupae (e.g., silkworm *Bombyx mori*). In contrast, when used in cultured insect cell systems, TGE offers the same advantages as BEVS while providing a faster and virus-free alternative suitable for rapid screening and early-stage development. Furthermore, TGE produces yields comparable or superior to BEVS (Korn et al., 2020; Lampinen et al., 2024). Despite the fact that insect cells produce native insect-type N-glycosylation patterns that differ from mammalian glycans, advances in glyco-engineering regarding the host cells or BEVS have expanded their applicability to functional antibody production for research, diagnostics, and emerging biopharmaceutical concepts. In the following sections, BEVS, as well as its advancements in glyco-engineering, and TGE will be discussed.

#### 2.6.1 Baculovirus

For over three decades, BEVS has served as an essential eukaryotic platform for the production of recombinant proteins, including functional antibodies and antibody fragments such as IgG, Fab and scFv (Smith et al., 1983; Zu Putlitz et al., 1990; Laroche et al., 1991; Reis et al., 1992; Abrams et al., 1994). The system relies on arthropod-specific recombinant baculoviruses, primarily the Autographa californica multiple nucleopolyhedrovirus (AcMNPV) and the less frequently used Bombyx mori nucleopolyhedrovirus (BmNPV), to efficiently infect insect cells, larvae or pupae (Motohashi et al., 2005; Hwang et al., 2024). Baculoviruses belong to the family of *Baculoviridae* and are enveloped, double-stranded DNA viruses that drive high protein expression by inserting the gene of interest into the nonessential polyhedrin (*polh*) locus, leveraging the strength of the very-late viral polh promoter. Alternatively, the very-late viral p10 promotor is also used for high levels of gene expression (Harrison et al., 1996; Shi and Jarvis, 2007; Grose et al., 2021). An overview of the antibodies produced with the BEVS system is shown in Table 7.

**TABLE 7:** Insect cells BEVS host reported for Monoclonal Antibody (mAb) expression.

Host	Antibody format	Antigen	Yield	Notes	References
Silkworm larvae	IgG	RBD	1.30 mg/10 mL hemolymph	Comparable affinity to mammalian-produced antibodies	Ebihara et al. (2021)
	Fab	RBD	1.44 mg/10 mL hemolymph		
	scFv (V_H_V_L_)	RBD	2.34 mg/10 mL hemolymph		
	scFv (V_L_V_H_)	RBD	2.64 mg/10 mL hemolymph		
Silkworm larvae	IgG (Mab-Bac)	HER2	4.9 mg/100 larvae	Native insect-type N-glycans	Egashira et al. (2018)
	IgG (mammalian-like N-glycans; Mab-BacG EP)	HER2	4.8 mg/100 larvae	Late E-vp39 promoter for heavy chain and very-late polh promoter for light chain; GlcNAc-terminated Fc glycans; increased ADCC activity	
	IgG (mammalian-like N-glycans; Mab-BacG PP)	HER2	3.1 mg/100 larvae	Very-late polh promoter for both chains; GlcNAc-terminated Fc glycans; increased ADCC activity	
Sf9 cells	IgG (H2L2)	HIV-1 gp120 protein	2–3 mg/L	Functionally equivalent to mammalian-produced antibody	Liu et al. (2014)
Sf9 cells	Chimeric IgE	CCDs	*>*30 mg/L	Biologically active despite insect-type N-glycans	Bantleon et al. (2016)

The successful production of CR3022 antibody in three formats (scFv, Fab and IgG) was demonstrated by Ebihara et al. using BEVS in silkworm. The monoclonal CR3022 antibody is a neutralizing antibody that targets the receptor binding domain (RBD) of the spike protein of SARS-CoV. This antibody has also demonstrated a strong affinity for the RBD of SARS-CoV-2. Here, the hemolymph was collected 4 days post-infection and 1.3 mg of IgG, 1.44 mg of Fab, 2.34 mg of scFv (V_H_V_L_) and 2.64 mg of scFv (V_L_V_H_) were purified from 10 mL of silkworm hemolymph. Notably, the resulting antibodies demonstrated antigen-binding affinity that was comparable to those produced in mammalian cells (Ebihara et al., 2021). In a separate study by Egashira et al., BEVS in silkworm was utilized to generate anti-HER2 (human epidermal growth factor receptor 2) IgGs in larvae containing native N-glycan patterns (Mab-Bac) and mammalian-like N-glycan patterns (Mab-BacG EP and Mab-BacG PP). The Mab-BacG EP and Mab-BacG PP constructs are distinguished by their promoter utilization for the heavy and light chain. Mab-BacG EP was engineered with the late E-vp39 promoter for the heavy and the very-late polh promoter for the light chain. In contrast, Mab-BacG PP was designed to express both chains using the very late polh promoter. The antibodies were produced for 6 days and subsequently quantified. With Mab-Bac 4.9 mg/100 silkworm larvae, with Mab-BacG EP 4.8 mg/100 silkworm larvae and with Mab-BacG PP 3.1 mg/100 silkworm larvae were produced. Notably, glycan analysis showed a shift towards mammalian-like N-acetylglucoseamine-terminated Fc glycans in Mab-BacG EP/PP, and the engineered antibodies exhibited increased ADCC activity (Egashira et al., 2018). Moving from *in vivo* systems to cell cultures, Liu et al. described the production of two HIV-1 broadly neutralizing H2L2 (IgG) antibodies, namely, b12 and VRC01, in Sf9 cell cultures. The production process for each antibody involved co-transducing Sf9 cells with separate recombinant baculoviruses encoding the light-chain and heavy-chain genes, respectively. A MOI of 1, or a co-transduction of two viruses at a MOI of 0.5 each, was used and the antibodies were harvested 3 days post-transduction. However, to optimize the production yield, the researchers implemented a co-infection strategy employing a 1:2 ratio of light chain to heavy chain baculoviruses. Using this strategy, the authors achieved purified antibody yields of 2–3 mg/L of insect cell culture for each antibody. Additionally, the authors validated that the insect-cell produced b12 antibody is functionally equivalent to the antibody produced in mammalian cell (Liu et al., 2014; Zhang Z et al., 2024). Extending the production capability to complex isotypes, the study of Bantleon et al. showed the functional and efficient production of a cross-reactive carbohydrate determinants-specific recombinant leporid/human chimeric IgE utilizing BEVS. The production of the IgE was conducted in 400 mL suspension cultures for a period of 5 days in Sf9 and Hi5 cells. The process yielded more than 30 mg/L of secreted IgE from Sf9 cells. In Hi5 cells, a lack of IgE was observed in the cell culture medium. Despite this, a significant amount of protein was detected within the cells of both cell lines. Additionally, the IgE produced in Sf9 cells retained high-affinity binding to its antigen, therapeutic anti-IgE and FcεRI, confirming biological activity even with the presence of insect type N-glycans (Bantleon et al., 2016).

Furthermore, advanced vector systems have been developed to overcome the native limitations of insect specific glycosylation, which is a critical factor regarding therapeutic glycoproteins. For example, MultiBac, which was originally designed for multi-subunit protein assembly, has been used to create specialized vectors such as SweetBac (Berger et al., 2004; Palmberger et al., 2012; Feng et al., 2024). SweetBac and related systems, such as PolyBac, incorporate e.g., mammalian glycosyltransferases directly into the baculovirus genome, thereby successfully producing mammalian-like N-glycans on antibodies and other complex proteins (Palmberger et al., 2012; Maghodia et al., 2021). An additional development was the refinement of the transposition-based system for producing recombinant baculoviruses, resulting in the creation of Bac-2-the-Future. This system encompasses optimized expression vectors, novel *E. coli* strains and enhanced protocols to significantly enhance the efficiency of the baculovirus production process (Mehalko and Esposito, 2016). Recent engineering advances also utilize CRISPR-Cas9 technologies for site-specific genome editing in the baculovirus-insect cell system (Mabashi-Asazuma and Jarvis, 2017). With this technology, limitations, like the integration of genes at random sites via nonhomologous recombination, can be overcome, and expanding the utility of BEVS. The application of these precise tools has facilitated the advancement of sophisticated host-cell engineering efforts that were previously constrained. Additionally, by integrating genes under the control of mammalian promoters, such as cytomegalovirus-originating ones, baculoviruses can be used to infect mammalian cells. This allows the virus, which is manufactured in insect cells, to deliver genetic cargo to mammalian cells for expression without reproducing itself (Mansouri and Berger, 2018).

Nevertheless, the use of BEVS for antibody production is associated with several inherent disadvantages. The baculovirus system itself is lytic and remodels the host cell, particularly the secretory pathway, resulting in lower antibody yields than in established mammalian platforms. Furthermore, this remodeling of the host cell can compromise antibody quality. The lytic nature of the virus renders the purification process more labor-intensive, as the culture medium becomes contaminated with substantial quantities of baculoviral and host-cell proteins as well as baculovirus itself. These limitations are extremely relevant during downstream manufacturing especially of recombinant viral vectors (rAVV) or Virus-like-particle. Complex purification processes have been set up to obtain pure material (Reiter et al., 2020; De Mathia et al., 2025; Lee et al., 2019) or complete baculovirus-free production in insect cells by plasmid-based expression is employed, as described in the next chapter.

#### 2.6.2 Plasmid-based transient gene expression in insect cells

Beside the conventional BEVS described above, a baculovirus-free, plasmid-based TGE method can be used to produce recombinant antibodies in insect cells. This system relies on direct plasmid transfection of *T. ni* Hi5 or *S. frugiperda* Sf9 cells typically using the cationic polymer polyethylenimine (PEI). In recent years, several protocols have been published with important modifications to optimize protein yield and transfection efficiency (Ogay et al., 2006; Shen et al., 2014; Shen et al., 2015; Bleckmann et al., 2019; Bleckmann et al., 2016; Bleckmann et al., 2015; Korn et al., 2020). An overview of the antibodies produced in insect cells with the TGE method is shown in Table 8.

**TABLE 8:** Plasmid-based insect cell host reported for Monoclonal Antibody (mAb) expression.

Host	Antibody format	Antigen	Yield	Notes	References
Hi5 cells	Fab	Bovine RNase A	∼120 mg/L	​	Mori et al. (2017)
Hi5 cells	hIgG	Botulinum toxin	∼80 mg/L	​	Korn et al. (2020)
Hi5 cells	mIgG	cMyc peptide	∼80 mg/L	​	Korn et al. (2020)
Hi5 cells	scFv-hFc	Several	60–100 mg/L	12 different scFvs-hFc were produced	Korn et al. (2020)
Hi5 cells	scFv-eqFc	Equine interleukin	46 mg/L	Showed no expression in HEK cells	Korn et al. (2020)

Although the TGE approach shows great potential, it remains relatively new, and only a few studies have investigated its use for antibody production. Mori et al. demonstrated efficient production of antibody Fab fragments in Hi5 cells, achieving yields of approximately 120 mg/L (Mori et al., 2017). In the study by Korn et al., IgG was expressed at around 80 mg/L, while scFv-Fc constructs reached yields between 50 and 100 mg/L. Remarkably, all 15 tested antibodies could be successfully produced using this baculovirus-free, transient insect cell system, including human, mouse and equine Fc versions. Notably, IgGs were producible in similar yields as in mammalian HEK Expi293F cells (Korn et al., 2020).

These yields are higher than those typically reported for BEVS (2–3 mg/L), which may be related to differences in expression efficiency between individual antibodies but can be also caused by the remodeling of the host cell secretion pathway during baculoviral infect (Zhang Z et al., 2024; Liu et al., 2014). In addition to the maintained cell viability, the plasmid-based approach offers a significantly simplified workflow, high reproducibility, and a very short cloning-to-protein turnaround of about 96 h compared to BEVS (Korn et al., 2020).

Overall, the baculovirus-free insect cell platform enables efficient, high-yield antibody production while retaining the ethical and regulatory benefits of animal-free production and the post-translational modification capabilities of a eukaryotic system, making it a promising platform for sustainable recombinant antibody manufacturing.

## 3 Antibody production in prokaryotic hosts

### 3.1 Gram-negative bacteria

Despite the rising number of recombinant protein expression systems, *E. coli* has retained its relevance as a cost-effective production system with rapid growth rates, high volumetric yields, and easy genetic manipulation (Rosano and Ceccarelli, 2014; Gupta and Shukla, 2017). Multiple *E. coli* strains have also achieved a GRAS classification. An overview of the antibodies produced in Gram-negative bacteria is shown in Table 9.

**TABLE 9:** Gram-negative bacterial host reported for Monoclonal Antibody (mAb) expression. N.D. not disclosed.

Host	Antibody format	Antigen	Yield	Notes	References
*Escherichia coli*	Fab	Matrix metalloproteinase-14 (MMP-14)	30 mg/L	Fed-batch fermentation with coexpression of DsbA/C	Rodriguez et al. (2017)
*E. coli*	F(ab')2	CD18	2,500 mg/L	Fermentation with protease- deficient strains	Chen et al. (2004)
*E. coli*	Fab	N.D.	2000–2,600 mg/L	Fed-batch fermentation with protease- deficient strains and coexpression of DsbC	Ellis et al. (2017)
*E. coli*	Nanobody	Nup93	116 mg/L	Cytoplasmic expression in SHuffle strain	Pleiner et al. (2015)
*E. coli*	scFv	HER2	147 mg/L	Cytoplasmic expression in SHuffle strain	Ahmadzadeh et al. (2020)
*E. coli*	scFv	α4-Integrin	139 mg/L	Shake flask using CyDisCo for cytoplasmic expression	Gaciarz et al. (2016)
*E. coli*	scFv	HER2	271 mg/L	Shake flask using CyDisCo for cytoplasmic expression	
*E. coli*	Fab	HER2	660 mg/L	Fed-batch fermentation using CyDisCo for cytoplasmic expression	Castillo-Corujo et al. (2024a)
*E. coli*	scFv	phOx	1,200 g/L	3 L fermentation	Sletta et al. (2004)
*E. coli*	scFv	EpCAM extracellular domain	112 mg/L	Optimal fermentation conditions and strains predicted with machine learning models	Hashemi et al. (2022)
*E. coli*	scFv	Omega peptide of β-galactosidase	420–650 mg/L	Fed-batch fermentation with ambr® 15f at 15 mL scale	Velez-Suberbie et al. (2018)
*E. coli*	scFv	Omega peptide of β-galactosidase	550–610 mg/L	Fed-batch fermentation in 1L fermenter	
*E. coli*	Fab	VEGF-A	347 mg/L	Fed-batch fermentation in 5L fermenter	Kim et al. (2018)
*E. coli*	Fab	VEGF-A	406 mg/L	Fed-batch fermentation in 50L fermenter	
*E. coli*	Fab_H3_	SARS-CoV-2 RBD	128–143 mg/L	20 mL shake flasks	Tungekar and Ruddock, (2023)
*E. coli*	nnAA-IgG	CD74	1,100 mg/L	high density fermentation	Blake-Hedges et al. (2024)
*Pseudomonas putida*	scFv	Lysozyme	1.5 mg/L	200 mL shake flask	Dammeyer et al. (2011)
*P. putida*	scFv	MUC1	3.6 mg/L	200 mL shake flask	
*P. putida*	scFv	CRP	2.9 mg/L	200 mL shake flask	
*Vibrio natriegens*	Nanobody	SARS-CoV-2 Spike	2.5–36.6 mg/L	2 L fermentation	Smith et al. (2024)

The first production of functional antibody fragments in *E. coli* was reported in 1988, where the secretion of the antibody chains into the oxidizing conditions of the periplasm enabled correct disulfide bond formation (Skerra and Plückthun, 1988; Better et al., 1988). The periplasm has since been established as the preferred compartment for recombinant antibody production in *E. coli*, with ongoing research into optimizing secretion (Kleiner-Grote et al., 2018; Rosano et al., 2019; Karyolaimos and de Gier, 2021). The translocation of antibody fragments into the periplasm is typically achieved with N-terminal leader peptides targeting the Sec or SRP (signal recognition particle) pathways (Thie et al., 2008). Frequently used leader peptides for the Sec pathway originate from the outer membrane protein A (OmpA), alkaline phosphatase (PhoA), and pectate lyase B (PelB) (Tachibana et al., 2004; Sletta et al., 2007). For the SRP pathway, leader peptides are used from the protein disulfide isomerase I (DsbA), the regulatory protein of torCAD (TorT), and TolB (Steiner et al., 2006; Thie et al., 2008; Lee and Jeong, 2013). While engineering attempts of leader peptides have yielded mixed results, modifying the leader peptide to regulate the translational strength (Kulmala et al., 2019; Mirzadeh et al., 2020) and increase the hydrophobicity (Zhou et al., 2016) has improved antibody production in individual cases. Additionally, tuning transcription levels not to exceed the capacity of the Sec-translocation apparatus has been shown to improve the periplasmic expression of model single-chain variable fragments (scFvs) (Schlegel et al., 2013; Baumgarten et al., 2018). The Tat-pathway has also been explored for the translocation of scFvs in a reduced yet folded form into the periplasm, where disulfide bond formation can occur (Alanen et al., 2015; Browning et al., 2017).

To support correct folding during recombinant antibody production, periplasmic chaperones such as FkpA, SurA, Skp (Friedrich et al., 2010; Sonoda et al., 2011; Wang et al., 2013; Shriver-Lake et al., 2017) and cytoplasmic chaperones like DnaKJE, GroESL, GrpE, and trigger factor TF (Hu et al., 2007; Dariushnejad et al., 2019) have been co- or overexpressed. The overexpression of the periplasmic enzymes responsible for correct disulfide bond formation, DsbA and DsbC, has also improved Fab (Fragment antigen-binding) assembly (Rodriguez et al., 2017). The periplasm of *E. coli* has been further optimized through strain engineering to prevent proteolytic degradation by eliminating periplasmatic proteases like Tsp and DegP (Chen et al., 2004; Aucamp et al., 2014). For high cell-density fermentation with Tsp-deficient strains, an additional mutation of the *spr* gene is necessary to restore cell viability (Chen et al., 2004). The combination of the Tsp-deficient strain with the stabilizing *spr* mutation and co-expression of DsbC enabled Fab yields up to 2.6 g/L in fermentation experiments (Ellis et al., 2017). Further studies have investigated the effects of modifying translation levels of various periplasmic chaperones and proteases on scFv production (Gawin et al., 2020).

The production of disulfide bond-containing proteins in the reducing environment of the cytoplasm leads mostly to protein aggregation and the formation of inclusion bodies (Wörn et al., 2000). While there have been advances in the recovery of functional proteins from inclusion bodies, the process can be time and therefore, cost intensive, leading to difficult upscaling (Martineau et al., 1998; Singh et al., 2015; Maggi and Scotti, 2017; Noguchi et al., 2017; Bhatwa et al., 2021). To overcome this limitation, different strategies to enable cytoplasmic disulfide bond formation have been developed. The first approach disrupts reducing pathways through mutations of the thioredoxin reductase (*trxB*) and glutathione reductase (*gor*) genes (Prinz et al., 1997; Bessette et al., 1999). This is frequently supplemented with the co-expression of signal sequence-less variant of the periplasmic disulfide isomerase DsbC (Levy et al., 2001; Jurado et al., 2002; Lobstein et al., 2012) or other cytoplasmic chaperones (Liu et al., 2019; Gaffar et al., 2024). The resulting origamiTM (Novagen) and SHuffle® (New England Biolabs) strains have been utilized for the cytoplasmic production of functional nanobodies (Knauf et al., 2023; Pleiner et al., 2015), scFvs (Heo et al., 2006; Ahmadzadeh et al., 2020; Wang et al., 2022), Fabs (Yusakul et al., 2018; Krittanai et al., 2020; Talaei et al., 2021), and aglycosylated IgGs (Robinson et al., 2015; Reddy et al., 2018; Robinson et al., 2023). The second strategy focuses on introducing catalysts for *de novo* disulfide bond formation into the cytoplasm of *E. coli*, without targeting the cytoplasmic reducing pathways. One version of this approach, CyDisCo (Cytoplasmic Disulfide bond formation in *E. coli*), utilizes the co-expression of the sulfhydryl oxidase Erv1p from *S. cerevisiae* and the human disulfide isomerase PDI (Hatahet et al., 2010; Nguyen et al., 2011). This system enabled the production of different functional scFvs and Fabs (Gaciarz et al., 2016; Gąciarz et al., 2017; Tungekar et al., 2023). The cytoplasmic production of functional Fab fragments was reported with yields of 565–660 mg/L in fed-batch fermentation experiments (Castillo-Corujo et al., 2024a). A direct comparison of the SHuffle and CyDisCo systems reported higher yields for scFv and Fab with the CyDisCo approach (Castillo-Corujo et al., 2024b). The CyDisCo system has also been combined with transport of folded and disulfide-bonded scFvs into the periplasm via the Tat-pathway (Matos et al., 2014; Alanen et al., 2015).

Recombinant antibodies are usually purified from either the periplasmic fraction or the cytoplasm of *E. coli*. There have been efforts to facilitate secretion into the culture medium, which has the advantage of reducing host protein and endotoxin contamination. However, extracellular expression is complicated by the outer membrane of Gram-negative bacteria (Baneyx and Mujacic, 2004; Lokireddy et al., 2025). The haemolysin secretion system can bypass the periplasm and has been used for the secretion of scFvs and variable domain heavy-chain-only antibody (VHH) into the culture medium (Fernández et al., 2000; Ruano-Gallego et al., 2019). The type II secretion system has been successfully targeted by the leader peptide STII for the extracellular expression of Fabs (Luo et al., 2019). The release of recombinant antibodies and other proteins into the culture medium can also be achieved by altering the permeability of the outer membrane through genetic engineering (Zhou et al., 2018; Gao et al., 2018) or adjusting cultivation conditions (Ukkonen et al., 2013; Voulgaris et al., 2015; Hsu et al., 2016). *E. coli* strains have also been engineered to simplify downstream protein processing, including the development of endotoxin-free strains (Mamat et al., 2015).

The production of recombinant antibodies in *E. coli* has also been improved by using genome integrated systems (Fink et al., 2019; Vazulka et al., 2022), strain engineering (McKenna et al., 2019; Landberg et al., 2020), adapting production rates through vector optimization, promoter choice and induction conditions (Baumgarten et al., 2018; Petrus et al., 2019; Karyolaimos et al., 2019) as well as optimizing cultivation parameters like temperature and media (Ukkonen et al., 2013; Hsu et al., 2016; Kim et al., 2018; Kumar et al., 2019). However, the optimal parameters for both upstream and downstream processes are heavily influenced by the individual antibody fragment (Hust et al., 2009; Farasat et al., 2017; Fink et al., 2021b). Machine learning models have also been applied to predict optimal induction conditions and *E. coli* strains for the production of soluble scFvs (Hashemi et al., 2022). High yields of recombinant antibodies are achieved through high cell-density fermentation, reaching g/L scale (Sletta et al., 2004; Aucamp et al., 2014; Ellis et al., 2017). Fermentation productions of antibodies have been performed at various scales, ranging from scaled-down bioreactors with volumes of microliters (Fink et al., 2019; Vazulka et al., 2022) or milliliters (Velez-Suberbie et al., 2018) to benchtop devices of several liters (Miethe et al., 2013; Voulgaris et al., 2015; Hsu et al., 2016) to larger laboratory bioreactors (Lindner et al., 2014; Kim et al., 2018; Fink et al., 2021a).

A major limitation in antibody production using *E. coli* is the absence of a human-like N-glycosylation machinery, restricting production to small antibody formats or aglycosylated IgGs. Current bacterial glycosylation approaches rely mainly on two-step hybrid systems, for example, the use of oligosaccharyltransferases to transfer bacterial-type glycans onto proteins or the incorporation of reactive aldehyde groups for glycan coupling, followed in both cases by chemo-enzymatic remodeling to human-type glycosylation *ex vivo* (Sotomayor et al., 2025; Smith et al., 2014). As these strategies are technically demanding, eukaryotic expression hosts still remain the preferred systems for producing antibodies with human-like glycosylations.

Besides common antibody formats, novel ones have been developed and efficiently produced in *E. coli*. For example, folding of disulfide-bonded fragments via the CyDisCo system enables good cytoplasmic production of a Fab_H3_ format, while another design called “Zipbody” with a leucine-zipper motif, improves chain pairing and solubility compared to standard Fabs in *E. coli* (Ojima-Kato et al., 2016; Tungekar and Ruddock, 2023). Moreover, the incorporation of non-canonical amino acids into antibody fragments and even full-length IgGs is possible, with titers above 1 g/L (Blake-Hedges et al., 2024). While demonstrating the first successful solution to the light chain pairing problem in the production of bispecific antibodies, dsFv-dsFv’ formats were only very poorly producible in *E.coli*, illustrating its limits in producing formats depending on more complex disulfide bridge patterns (Schmiedl et al., 2000).

Other Gram-negative expression hosts have also been explored for antibody production. *Proteus mirabilis* L-forms were shown to produce miniAbs and scFvs (Kujau et al., 1998; Rippmann et al., 1998) and *Pseudomonas putida* has likewise been used to express scFvs (Dammeyer et al., 2011). More recently, small scale production in shake flasks of certain nanobodies in *Vibrio natriegens* surpassed the yields obtained in *E. coli*, showing that nanobodies can also be expressed in alternative Gram-negative hosts (Smith et al., 2024). However, Gram-negative systems other than *E. coli* have not progressed beyond proof of concept for antibody production and available studies remain very limited.

### 3.2 Gram-positive bacteria

Another established option for the industrial production of proteins are Gram-positive bacteria. They not only share advantages like a high growth rate and simple cultivation with Gram-negative bacteria but also have an efficient secretory pathway. To date the production of antibodies in Gram-positive bacteria is still limited to smaller antibody fragments. While numerous reports for the production of scFv, VHH, Fab and scFab antibodies have been reported (Yang et al., 2020; Ye et al., 2011; Jordan et al., 2007a; Jordan et al., 2007b; Kes et al., 2025; Mizukami et al., 2018), the production of full size IgGs remains an unsolved problem. However there have been reports for low level expression of an scFv-Fc which failed to be secreted into the culture medium (Schilling et al., 2025). An overview of the antibodies produced in Gram-positive bacteria is shown in Table 10.

**TABLE 10:** Gram-positive bacterial host reported for monoclonal antibody (mAb) expression.

Host	Antibody format	Antigen	Yield	Notes	References
*B. subtilis WB800N*	scFv (D1.3)	Lysozyme	130 mg/L	Cultivated in microtiter plate (1,250 µL scale) and quantified by ELISA	Lakowitz et al. (2017)
*B. licheniformis*	scFv (D1.3)	Lysozyme	17 mg/L		
*P. megaterium*	scFv (D1.3)	Lysozyme	15 mg/L		
*B. choshinensis*	Fab (Trastuzumab)	HER2	1,200 mg/L	Fed-Batch cultivation, quantified by SDS-PAGE	Mizukami et al. (2018)
*B. choshinensis*	VHH	IZUMO1	3,000 mg/L		Mizukami et al. (2015)
*Bifidobacterium longum*	scFv, VHH	TNF-α, *C. difficile* exotoxine A	25 μg/L, 0.3–1 mg/L	Lab-scale production, antibody fragments show biological activity	Shkoporov et al. (2015)
*Corynebacterium glutamicum*	scFv	Anthrax toxin	68 mg/L	Fed-Batch cultivation in 5-L lab scale bioreactor	Yim et al. (2014)
*Lactococcus lactis*	scFv	CTLA-4	n.d	2 mL scale expression, activity tested via Western blot and ELISA	Namai et al. (2020)

Advantages of Gram-positive expression systems are the lack of an outer membrane and their Lipopolysaccharide-free cell wall. This allows for efficient secretion into the culture medium, reducing downstream processing and enabling an endotoxin-free production (Tjalsma et al., 2000; Tjalsma et al., 2004). A significant portion of *Bacillus* and *Lactobacillus* strains have been classified as GRAS, as they are widely used for food production. Furthermore, the secretion of antibody fragments into the extracellular environment via the secretion-dependent pathway results in antibody fragments with their native structure and disulfide bonds. This process prevents the formation of inclusion bodies (Tjalsma et al., 2004; Schilling et al., 2025) which is frequently observed in the periplasmic space of Gram-negative bacteria which mandates time-consuming and very low yield refolding methods.

The major production systems for these antibody fragments are *Bacillus subtilis* and *Priestia megaterium* (previously *B. megaterium)* (Lakowitz et al., 2017; Jordan et al., 2007a; Lüders et al., 2011). The expression of antibody fragments in other *Bacillus* species with industrial application has been reported for *B. brevis*, *B. licheniformis* and *Brevibacillus choshinensis* (previously *B. choshinensis)* (Mizukami et al., 2018; Lakowitz et al., 2017; Inoue et al., 1997). Successful productions were also reported in other species including *Bifidobacterium longum* (Shkoporov et al., 2015), *Corynebacterium glutamicum* (Liu et al., 2016; An et al., 2013; Yim et al., 2014)*, Lactococcus lactis* (Namai et al., 2020; Ravnikar et al., 2010) and numerous *Lactobacillus* strains (Marcobal et al., 2016; Pant et al., 2006; Martín et al., 2011). Furthermore multiple *Staphylococcus* strains were successful in displaying antibody fragments on their cell surface (Cavallari, 2017; Yoon et al., 2024; Gunneriusson et al., 1996).

In recent years, a steady increase in yields for *B. subtilis* and *P. megaterium* could be observed. The *B. subtilis* system has emerged as a competitive alternative to the established *E. coli* systems for the production of antibody fragments. Due to its role as a model organism for bacterial devolvement and its status in industrial biotechnology, *B. subtilis* has become one of the most studied bacteria (Stülke et al., 2023). Many different optimizations have led to an increase in antibody yield. A major step was the development of multiple protease-deficient strains with reduced genome size. The total genome size was reduced from 4.2 Mb to as low as 2.5 Mb for the PS38 strain (Reuß et al., 2017). The amount of expressed and secreted protein increased steadily with each reduction in genome size. Furthermore the secretion of a difficult-to-express protein with five disulfide bonds increased by up to 3000-fold compared to the reference strain (Schilling et al., 2023; Michalik et al., 2021). Other optimization strategies that aimed to increase overall productivity include co-expression of chaperones and other helper proteins. Additional chaperones could increase the secretory scFv yield by up to 60% (Wu et al., 1998; Wu et al., 2002). Screening and designing different signal peptides and promoters could further improve the protein expression and secretion (Gao et al., 2025; Yoon et al., 2024; Heinrich et al., 2019).

In addition to the specific antibody and its molecular format, the quantity of secreted antibody depends on various cultivation parameters. The highest reported yields are ∼1,200 mg/L for the trastuzumab Fab and ∼3,000 mg/L for Anti-IZUMO1 VHH, both of which were expressed in *B. choshinensis* using fed-Batch cultivation (Mizukami et al., 2018; 2015). These yields were calculated by measuring the protein band intensities on an SDS-PAGE with BSA as a standard. This method may have overestimated the yield by including impurities of the same molecular weight and nonfunctional antibody fragments. Comparable yields have not been reproduced in other publications. The anti-Lysozym D1.3 scFv has been used as a model antibody in multiple studies reaching yields of up to 130 mg/L of secreted scFv in the *B. subtilis* medium. Reporting the third highest yield published for Gram-positive bacteria (Lakowitz et al., 2017). This result was quantified by ELISA measurements and therefore only includes functionally binding scFvs for quantification. Various reports achieved yields up to 15 mg/L for *P. megaterium* using the same antibody. This is more than a 35-fold increase in comparison to previous publications for *P. megaterium* (Schilling et al., 2025; David et al., 2011; Jordan et al., 2007a; Jordan et al., 2007b; Lüders et al., 2011).

Today the antibody production in Gram-positive bacteria like *B. subtilis* is becoming a competitive alternative to the *E. coli* expression system, although it is still limited to antibody fragments. Even though antibody yields are lower, Gram-positive bacteria show improved antibody folding and low downstream processing costs. The GRAS classification of various Gram-positive bacteria also allows for a unique proof-of-concept application currently under investigation: living biotherapeutics, including an anti-IgE scFv-expressing *Lactobacillus* for direct mucosal immunization or vaginal colonizing Lactobacilli expressing anti-HIV-1 scFvs to prevent virus transmission (Scheppler et al., 2005; Marcobal et al., 2016). Other applications utilize the highly resistant spores produced by *B. subtilis* to store VHHs under extreme environments (Yang et al., 2020).

## 4 Transgenic plants

The production of recombinant proteins in transgenic plants has now been explored and investigated for over 30 years. The first examples of therapeutic proteins produced in plants date back to the late 80s, where first the human growth hormone was recombinantly expressed in tobacco and sunflower cells (Barta et al., 1986). Soon after an antibody was expressed in tobacco plants (Hiatt et al., 1989). This approach, encompassed under the term “molecular pharming”, explores the possibility to produce large amounts of therapeutic proteins in plants that require minimal energy sources (in principle only light and water) at a fraction of the cost typically necessary for mammalian production systems. Furthermore, plants do not share pathogens with humans, can conduct complex post-translation modifications (formation of disulfide bridges, assembly of complex multimeric complexes and glycosylation) similar to other higher eukaryotes and exhibit considerable advantages in terms of up-scaling (Daniell et al., 2001; Obembe et al., 2011; Yusibov et al., 2016; Donini and Marusic, 2019). An illustrative, non-exhaustive list of antibodies expressed in plants is reported in Table 11.

**TABLE 11:** Transgenic plants host reported for Monoclonal Antibody (mAb) expression. Agrotransformation and agroinfiltration are mediated by *Agrobacterium tumefaciences*. Yields are expressed as antibody quantity per kg of fresh plant tissue (mainly leaves). n.a. not applicable.

Host	Antibody format	Antigen	Yield	Notes	References
*Nicotiana tabacum*	scFv	Botulinum toxin serotype A (BoNT/A)	20–40 mg/kg	Agrotransformation	Almquist et al. (2006)
*N. benthamiana*	IgG	HIV gp120 protein	105.1 mg/kg	Agroinfiltration, plantibody of HIV-neutralizing mAb 2G12	Sainsbury et al. (2010)
*N. benthamiana*	IgG and scFv-CH	West Nile virus (WNV) E protein	0.74–0.77 g/kg	Agroinfiltration, plantibody of WNV-neutralizing mAb E16	Lai et al. (2014), Oliphant et al. (2005), He et al. (2014)
*N. benthamiana*	IgG	Ebola virus GP1 protein	0.4–0.5 g/kg	Agrotransformation	Huang et al. (2010)
*N. tabacum*	scFv-RNase	Hepatocellular carcinoma	0.75–1.99 mg/kg	Agrotransformation	Cui et al. (2012)
*N. benthamiana*	decoy angiotensin-converting enzyme 2 fused to human Fc of IgG1 (ACE2-hFc)	n.a.	50 mg/kg (after preparative gel filtration chromatography)	Agrotransformation	Castilho et al. (2021)
*N. benthamiana*	human IgGs	SARS-CoV-2 Spike protein	4–35 mg/kg	Agroinfiltration	Shanmugaraj et al. (2020)
*N. benthamiana*	sectretory human IgA1 and IgA2	Enterotoxigenic *Escherichia coli* (ETEC) CfaE	1.1-8.7 mg/kg	Agroinfiltration	Teh et al. (2021)

The possibility of obtaining exogenous proteins from plant cells and tissues has been enabled by the crucial discovery of the natural mechanism of agro-infiltration/-transformation. The transfer of foreign genetic material to the nucleus of plant cells is mediated by *Agrobacterium tumefaciens* infection. The gene of interest is encoded by transfer DNA that is flanked by imperfect repeats on a binary vector, which allows replication both in *E. coli* and *A. tumefaciens* (Hoekema et al., 1983). Agro-transformation enables stable transfection and is induced mainly through biolistic approaches, or particle bombardment, where DNA is microprojected into plant cells (Klein et al., 1987), or through the infection of *A. tumefaciens*. A wealth of possibilities has been explored, reviewed elsewhere (Bélanger et al., 2024), but all require selection of the progeny or, more traditionally, crossings to obtain pure homozygous lines. On the other hand, transient transformation mediated by *A. tumefaciens*, termed agro-infiltration, is now established for both small- and large-scale productions, where transformed bacterial suspensions are applied to the underside of the leaves with a syringe, in the former case, or by vacuum infiltration, for large-scale approaches. These transient techniques, together with a constant research effort on vector improvement, allowed several companies to establish quick pipelines for therapeutics production in the order of grams of recombinant protein per kg of leaf material (Eidenberger et al., 2023). Furthermore, this has also allowed the production of reagents for research application (e.g., products from companies like Leaf Expression Systems and iBio, Inc.) (Eidenberger et al., 2023).

Regarding expression hosts, a wealth of species has been investigated to obtain recombinant protein and antibody expression in any plant organ (Twyman et al., 2003), but the most successful and employed model system is *Nicothiana benthamiana*, a wild relative of tobacco, given its excellent amenability to agro-infiltration and very well consolidated methods (Eidenberger et al., 2023). The tobacco plant, *N. tabacum*, has also been researched intensively for antibody and antibody fragment production (Ramírez et al., 2003; Schillberg et al., 1999; Düring et al., 1990; Ma et al., 2015). Other species have been harnessed for antibody production, e.g., alfalfa (*Medicago sativa*) (Bardor et al., 2003), the duckweed *Lemna minor* (Woodard et al., 2009; Barros et al., 2011; Naik et al., 2012), rice cells (Torres et al., 1999), *Arabidopsis thaliana* seeds (De Greve, 2022; De Wilde et al., 2013; De Buck et al., 2012), maize (Ramessar et al., 2008; Rademacher et al., 2008) and lettuce (He et al., 2012). The choice of expression system is always protein- and context-dependent and there is never a one-fit-for-all solution (Schütz et al., 2023). Indeed, the comparative analysis conducted by Stöger and colleagues nicely demonstrated the production of an anti-CEACAM antibody fragment, scFv T84.66, in different plant species, namely, tobacco, rice, wheat, pea and tomato, and organs thereof (Stoger et al., 2002). The antibody *per se* was producible to similar levels in all species (average yields between 0.1 and 30 g/kg tissue or organ), the main difference being the stability of the protein itself in different systems.

A variety of formats of plantibodies, i.e., antibodies expressed in plants, have been produced, ranging from scFv to multimeric complexes and immunoconjugates, both with therapeutic and diagnostic aims. Antibody 2G12, a neutralizing anti-HIV mAb, was indeed produced as a secretory dimeric IgA, a heterodecameric complex, in both transgenic tobacco plants and *N. benthamiana* transient system. The secretory IgAs proved to be correctly assembled and neutralized highly pathogenic HIV variants (Paul et al., 2014). Similarly, pentameric and hexameric IgM were successfully produced in the transient *N. benthamiana* system, properly folded and also glycosylated (Loos et al., 2014). Furthermore, immunocytokines, i.e., antibody moieties fused to cytokines, were also investigated for production in plants: pembrolizumab fused to IL15Ralfa-IL15 (Rattanapisit et al., 2025) and rituximab fused to IL2 (Marusic et al., 2016) were obtained via transient agro-infiltration in *N. benthamiana* with modest yields (8.8 mg/kg and 15–20 mg/kg leaves, respectively). The activity of the immunocytokines was demonstrated in mice or *in vitro* experiments, respectively. A further example of an immunoconjugate was developed for the fast and easy-to-use detection of aflatoxin M1 by fusing a monoclonal antibody specific for the toxin to two different fluorescent proteins. The fluorescent immunodiagnostic plantibodies were indeed successful in the detection and visualisation of aflatoxin in two different set-ups (Sterpa et al., 2025).

A crucial point in antibody therapeutics is the presence, extent and quality of glycosylation, responsible for several Fc-mediated functions, mainly interaction with FcγR and C1q (Jefferis, 2009). Although mammals and plants share the same core N-glycosylation residues (mannose and N-acetylglucosamine), the terminal residues are different: the former harbour β1,4-galactose and sialic acid antennary residues, whereas the latter contain terminal bisecting α1,3-fucose and core β1,2-xylose sugars (Gomord et al., 2010). Furthermore, O-glycosylation patterns differ: plants produce extensin- and arabinogalactan-type O-linked glycans, instead mammals synthesize O-linked glycans rich in mucin (Gomord et al., 2010). These differences can lead to differences in effector functions, pharmacokinetics and immunogenicity of the therapeutic antibody. Thus, several strategies have been devised to “humanize” glycosylation in plants, where either the full set of genes leading to mammalian terminal sugar residue synthesis and addition have been overexpressed (Castilho et al., 2010) or the plant-specific glycosylation pathway leading to α1,3-fucose and xylose glycosylation were knocked-out via CRISPR-Cas9 (Jansing et al., 2019). In both cases, antibodies against HIV gp41 (2G12 and 4E10) were successfully produced with the desired glycosylation pattern and demonstrated similar or even better IC_50_ compared to the standard CHO-produced one (Strasser et al., 2009). A more recent work investigated the production of the anti-CD20 antibody rituximab in agro-infiltrated *N. benthamiana* (Tengattini et al., 2025). With a yield of 50–75 mg/kg fresh leaves, rituximab could be glyco-engineered reducing plant-specific glycosylation patterns by either adding a mannosidase inhibitor during agro-infiltration or using previously described genome-modified plants (Jansing et al., 2019). Furthermore, the interaction with FcγRIIIa could be demonstrated (Tengattini et al., 2025).

As testimony to the efficacy and safety of plantibodies, clinical trials and use of antibody therapeutics produced in plants have been successful. The company Mapp Biopharmaceutical, Inc. developed ZMapp, a three-mAb cocktail neutralizing Ebola virus, transiently produced in *N. benthamiana* in a very rapid fashion, that showed preclinical efficacy in primates (Qiu et al., 2014; Zeitlin et al., 2011). Even though safety testing in clinical trials had not been conducted, the FDA granted an emergency investigational new drug approval of this cocktail during the 2014–2015 Ebola outbreak in West Africa and patients showed improved clinical parameters upon treatment and survived a typically lethal infection (Lyon et al., 2014). Other successful examples of clinical translation of plantibodies are represented by CaroRX, a secretory IgA/IgG targeting streptococcal antigen I/II of *Streptococcus mutans*, developed to prevent tooth decay and produced in tobacco plants (Ma et al., 1994; Ma et al., 1998). After initial successful testing, a larger Phase II randomized trial established no long term protective efficacy, showing however that the product was safe (Weintraub et al., 2005). Furthermore, scFvs were expressed in *N. benthamiana* to produce idiotype-specific antibodies to assemble a personalized vaccine against follicular B-cell (non-Hodgkin) lymphoma that was tested in a phase I clinical trial (McCormick et al., 2008), where almost half of the subjects did develop an antigen specific response. Finally, anti-HIV neutralizing mAb 2G12 has been used extensively in plant production systems as discussed above and was also tested in clinical phase I (NCT02923999) sponsored by FuturePharma, where safety of 2G12 produced in tobacco transgenic plants was confirmed (Ma et al., 2015). To the best of our knowledge, no further progress has been made as of December 2025.

Despite the great potential and successful first examples of clinical translation, plantibodies are still far from playing a major role in therapy, diagnostics and reagents for research areas. Unfortunately, cost of goods for initial biomass production is today only a small fraction of total development cost of a therapeutic antibody, therefore this potential advantage is offset by a number of uncertainties originating from non-standard purification and downstream processes, unknown regulatory hurdles and acceptance and less optimized systems, resulting in a very limited clinical translation of plantibodies.

## 5 Cell-free production systems

In cell-free systems for protein synthesis the transcription and translation machinery from crude cell extracts is used for protein production. The extract is prepared from living cells and contains ribosomes, translation factors and enzymes. For *in vitro* use, nucleotides, amino acids, energy sources, cofactors, buffers and salts are supplemented and template DNA (plasmid or PCR fragment) is added (Stech et al., 2013a; Garenne et al., 2021).

Protein synthesis in cell-free systems is disconnected from cell viability and enables an efficient, flexible and rapid production compared to cell-based systems (Dondapati et al., 2020). The process can easily be automated, enabling high throughput expression and screening (Beebe et al., 2014; Georgi et al., 2016). The open nature of the systems allows for control and adjustment of reaction conditions. This is especially important for the synthesis of antibodies and antibody fragments, as they require appropriate conditions for the formation of inter- and intramolecular disulfide bonds (Glockshuber et al., 1992; Goto and Hamaguchi, 1979). Furthermore, novel modified molecules can be constructed including bispecific antibodies and antibody drug conjugates (ADCs), as non-canonical amino acids can be incorporated in a site-specific manner (Xu et al., 2015; Zimmerman et al., 2014; Axup et al., 2012; Krebs et al., 2022b; Kanter et al., 2007; Patel et al., 2011). Therefore different approaches for antibody production in prokaryotic and eukaryotic systems emerged. Among prokaryotic systems, extracts based on *E. coli* have been so far applied for the synthesis of antibodies. Eukaryotic systems include extracts from CHO cells, wheat germ and insect cells. An overview of the antibodies produced in cell-free systems is shown in Table 12.

**TABLE 12:** Cell-free production systems reported for Monoclonal Antibody (mAb) expression. N.d.: not disclosed.

Host	Antibody format	Antigen	Yield	Notes	References
*E. coli* S30 extract	scFv	hemagglutinin	8 mg/L	First scFv expressed in *E. Coli* CFS	Ryabova et al. (1997)
*E. coli* S30 extract	scFv	lysozyme	30 mg/L	Both arrangements of vH and vL	Merk et al. (1999)
RTS 100 *E. coli* kit (Roche *Diagnostics)*	scFv	γ-seminoprotein	0.8 mg/mL	Active and specific, direction of magnetic beads to prostate cancer cells	Han et al. (2006)
RTS 100 *E. coli* kit (Roche *Diagnostics)*	scFv	ErbB-2	200 mg/L	​	Galeffi et al. (2006)
*E. coli* S30 extract	scFv	IL-23	500–950 mg/L	Linear scalable yields	Yin et al. (2012)
	Fab	IL13α1R	300 mg/L		
	IgG	HER2	400 mg/L		
*E. coli* S30 extract	scFv	HER2	>1,000 mg/L	Reoptimized protocol for simplified production	Cai et al. (2015)
	scFv-Fc	HER2	>500 mg/L		
	Fab	HER2	>1,500 mg/L		
	IgG	HER2	>500 mg/L		
	IgG	CD30	<500 mg/L		
*E. coli* S30 extract	scFv	EGFR	∼1 mg/mL	Crystal structure in complex with extracellular domain of human EGFR obtained (for scFv) Incorporation of non-natural amino acids shown Linear, scalable yields.	Matsuda et al. (2018)
	Fab	EGFR	∼1 mg/mL		
*E. coli* S30 extract	Fab	Catalytic activity	n.d.	Antibody 6D9, binding and catalytic activity	Jiang et al. (2002)
*E. coli* S30 extract	scFv	HSA	n.d.	Extract from protease deficient mutant (ΔdegP-ompT) of *E. coli* BW25113	Ali et al. (2005)
	Fab	Catalytic activity	20 mg/L		
*E. coli* S30 extract	Fab	BoNT/B	∼30 mg/L	Binding and neutralization.	Oh et al. (2010)
*E. coli* S30 extract	sdFab	SARS-CoV-2 (S6P, RBD)	n.d.	More uniform assembly compared to Fabs	Hunt et al. (2023)
	IgG	HER2	n.d.	Incomplete Assembly	
PURE system (GeneFrontier, Kashiwa, Japan)	sdFab Zipbody	*Vibrio parahaemolyticus*	30 mg/L	Development of a mAb screening system	Ojima-Kato et al. (2017)
		*E. coli* O26	n.d.		
*E. coli* S30 extract	sdFab Zipbody	*E. coli* O157	n.d.	​	Ojima-Kato et al. (2016)
*E. coli* S30 extract	IgG	Human creatine kinase	∼0.5 mg/L	Active, correctly folded and disulfide bridged	Frey et al. (2008)
*E. coli* S30 extract	IgG	HER2	1 g/L	Development of a DsbC and FkpA-overexpressing strain for extract production	Groff et al. (2014)
		CD30	1 g/L		
PURE system (GeneFrontier, Kashiwa, Japan)	IgG	HER2	124 mg/L	Synthesis temperature and template DNA ratio (Hc/Lc) was optimized	Murakami et al. (2019)
	IgG1, IgG2, IgG4 subclasses	​	33–73 mg/L		
Wheat germ extract	scFv	*Salmonella* O-antigen	13 mg/L	DTT deficient extract supplemented with PDI	Kawasaki et al. (2003)
tabacco BY-2 cell lysate	IgG	vitronectin	150 mg/L	Microsome-enriched lysates, IgG produced with melittin signal peptide for translocation	Buntru et al. (2015)
*Spodoptera frugiperda* (Sf21) cell lysate	scFv	fluorescein	20 mg/L	Microsome containing lysates, scFv produced with melittin signal peptide for translocation and enrichment in microsomes	Stech et al. (2013b)
*Spodoptera frugiperda* (Sf21) cell lysate	scFv	SMAD2	10–15 mg/L	Microsome- containing lysates, scFv produced with melittin signal peptide for translocation and enrichment in microsomes	Stech et al. (2014)
		SMAD2-P			
		CXCR5			
*Spodoptera frugiperda* (Sf21) cell lysate	Fab	CD4	10 mg/L	Microsome- containing lysates, Fab produced with melittin signal peptide for translocation and enrichment in microsomes	Merk et al. (2012)
		Lysozyme			
*Spodoptera frugiperda* (Sf21) cell lysate	Fab	Lysozyme	165 mg/L	Continuous Exchange Cell-Free-based reaction	Merk et al. (2015)
CHO cell lysate	scFv	SMAD2-P	n.d.	Continuous Exchange Cell-Free-based reaction, Microsome- containing lysates	Thoring et al. (2017)
CHO cell lysate	scFv-Fc	SMAD2-P	500 mg/L	Continuous Exchange Cell-Free-based reaction, Microsome- containing lysates	Stech et al. (2017)
	IgG	SMAD2-P	250 mg/L		
CHO High-Yield IVT Kit (Thermo Scientific, West Palm Beach, FL)	IgG	4-hydroxy-3-iodo-5-nitrophenylacetyl	58–114 mg/L	Light chain expressed before heavy chain template was added	Martin et al. (2017)
CHO cell lysate	Nb	EGFR	12 mg/L	Microsome- containing lysates	Haueis et al. (2022)
		GFP	<4 mg/L		

In the native reducing environment of the bacterial cytoplasm, disulfide bond formation cannot take place. Various strategies have been applied to enable *in vitro* formation of disulfide bonds for protein synthesis in cell-free systems. These strategies include adjustment of the redox potential by addition of reduced (GSH) and oxidized glutathione (GSSG) (Ryabova et al., 1997; Merk et al., 1999; Jiang et al., 2002; Kim and Swartz, 2004; Yin and Swartz, 2004), inactivation of endogenous reductases via pretreatment with alkylating agents like iodoacetamide (IAM) (Kim and Swartz, 2004; Yin and Swartz, 2004), engineering of strains without glutathione reductase genes *gor* and *trxB* (Knapp et al., 2007), addition of enzymes like PDI (Ryabova et al., 1997; Merk et al., 1999; Jiang et al., 2002) or *E. coli* DsbC (Zawada et al., 2011; Yin and Swartz, 2004; Kim and Swartz, 2004) and addition of chaperons (e.g., DnaK, DnaJ, GroEL, GroES) (Ryabova et al., 1997; Jiang et al., 2002; Merk et al., 1999).

The production of an scFv in an *E. coli* cell-free system was first reported by Ryabove et al., in 1997. The scFv was derived from the anti-hemagglutinin antibody 17/9 (Schulze-Gahmen et al., 1993). In the presence of PDI, chaperones and reduced and oxidized glutathione, 8 mg/L scFv could be produced in 15 min and about 50% of the antibodies were active (Ryabova et al., 1997). The development of *in vitro* transcription-translation platforms enabled the use of ribosome display for *in vitro* evolution and high-throughput screening (Hanes and Plückthun, 1997). In 1999 two HyHEL10 scFvs were produced in an *E. coli* cell-free system by Merk et al. Synthesis was conducted in the presence of PDI, chaperones, reduced and oxidized glutathione (Merk et al., 1999). Various scFvs were produced by other groups (Han et al., 2006; Galeffi et al., 2006; Yin et al., 2012; Cai et al., 2015), with yields >1 g/L (Cai et al., 2015).

Matsuda et al. (2018) expanded the capabilities of *E. coli* cell-free synthesis by showing that it can produce the first functional, fully human antibody fragments (scFv and Fab) targeting EGFR. By incorporating DsbC, reduced and oxidized glutathione, the reaction yielded 1 g/L within 7 h. Importantly, this work provided the first evidence that antibody fragments produced in a cell-free system are suitable for structural studies such as X-ray crystallography, enabling precise epitope characterization. Furthermore, some alternative compositions with lower substrate costs for the cell-free reaction for disulfide bonds have been exploited (Knapp et al., 2007; Wu Y. et al., 2022; Cai et al., 2015).

Jiang et al. were the first to establish that Fab fragments could be synthesized in a cell-free format, co-expressing both chains of the catalytic antibody 6D9 from dual templates (Jiang et al., 2002). The parental antibody 6D9 catalyzes the hydrolysis of a non-bioactive chloramphenicol monoester derivative to the antibiotic chloramphenicol (Miyashita et al., 1993; Miyashita et al., 1997). Their work highlighted the importance of folding-assistance components: supplementation with PDI, chaperones, and a glutathione redox pair improved Fab solubility and antigen-binding activity (Jiang et al., 2002). Subsequent improvements addressed proteolysis derived from endogenous serine proteases, a major yield-limiting factor. By generating a double protease-deficient *E. coli* strain (ΔdegP-ompT) for extract preparation, Ali et al. achieved substantially higher yields of both 6D9 Fab and an anti-HSA scFv, reaching ∼20 mg/L of 6D9 Ali et al. (2005). Further optimization, particularly the pretreatment of the extract with oxidized glutathione, enabled even greater productivity, such as the ∼30 mg/L production of the Fab fragment of anti-botulinum neurotoxin serotype B (BoNT/B) antibody BCXRH1 (Oh et al., 2010). A key mechanistic improvement was introduced by Yin et al. It was observed that the heavy chain alone is prone to aggregation and degradation. By temporally staggering expression, producing the light chain first, followed by addition of the heavy-chain template, they achieved a yield of 300 mg/L of an anti-IL13α1R Yin et al. (2012). An even higher yield was determined for a Fab derived from trastuzumab, over 1,500 mg/L were produced in a conventional cell-free system, as well as in an optimized cost-efficient system (Cai et al., 2015). Beyond classical Fab formats, synthetically dimerized Fabs (sdFabs) were successfully produced in *E. coli* cell-free systems (Ojima-Kato et al., 2017) and their assembly was more uniform when compared to the production in Fab format (Ojima-Kato et al., 2016; Hunt et al., 2023).

The first successful production of a full IgG in a prokaryotic cell-free system was reported by Frey et al., who synthesized the mouse monoclonal antibody MAK33. It was found that the presence of PDI or DsbC is crucial for functional antibody assembly, with either one around 0.5 mg/L active antibody being produced Frey et al., 2008). Yin et al. could produce 400 mg/L of trastuzumab (Yin et al., 2012). Later on, Groff et al. improved the production of trastuzumab by the addition of DsbC and the prolyl isomerase FkpA and achieved Gram per liter titers. To streamline production, they engineered an *E. coli* strain overexpressing DsbC and FkpA directly in the extract source, enabling consistently high titers across multiple Groff et al. (2014). Trastuzumab and other IgGs were also produced in the PURE (Protein synthesis Using Recombinant Elements) system with yields from 33 to 124 mg/L (Murakami et al., 2019).

Prokaryotic-based cell-free systems are cost effective, but when post-translational modifications are required or there are constraints regarding associated endotoxins, it is often necessary to transition to eukaryotic cell-free systems. For the production of antibodies and antibody fragments in eukaryotic cell-free extracts various systems have been employed. Animal-free systems include wheat germ extract, tobacco BY-2 cell lysate, Sf21 cell lysate and CHO extract.

The production of a scFv in a cell-free wheat germ system was first reported by Kawasaki et al. (2003). For synthesizing the scFv targeting the *Salmonella* O-antigen, the reducing agent dithiothreitol (DTT) had to be removed from the reaction, and the resulting DTT-deficient extract was supplemented with PDI. Half of the soluble fraction was active, 13 mg/L of functional scFv were synthesized (Kawasaki et al., 2003). A tobacco BY-2 cell-lysate-based cell-free system was the first eukaryotic platform shown to support IgG production. In this extract, remnants of the endoplasmic reticulum reassemble into microsomal vesicles that can receive proteins via melittin signal peptide-mediated targeting, enabling post-translational processing. Using a microsome-enriched preparation, IgG yields of 150 mg/L were achieved (Buntru et al., 2015).

A comparable microsome-targeting strategy was implemented in an Sf21 cell-derived cell-free expression system. Using this platform, an scFv based on the anti-fluorescein antibody B13-DE1 was synthesized, and its successful translocation into microsomal vesicles was demonstrated. Proper redox conditions were essential for obtaining functional protein, resulting in yields of 20 mg/L - representing the first report of scFv production in this system (Stech et al., 2013b). Subsequent studies extended this approach to additional scFvs, reaching titers of 10–15 mg/L (Stech et al., 2014). The Sf21 cell-free system also supported the generation of Fab fragments, as shown for Fabs (an anti-CD4 and an anti-lysozyme) produced at about 10 mg/L, with protein activity influenced by redox balance and microsomal translocation (Merk et al., 2012). Yield improvements were later realized using a Continuous Exchange Cell-Free (CECF) configuration, enabling synthesis of 165 mg/L of an anti-lysozyme Fab (Merk et al., 2015). The CECF system was also used for the production of scFvs in a CHO-based cell-free system, with superior yields of functional scFvs compared to batch reaction. Furthermore, the positive impact of the translocation into microsomes for functional scFvs was shown, as previously described for other cell-free systems (Thoring et al., 2017). This CHO cell-free system was also used for the synthesis of an IgG and a scFv-Fc by Stech et al. Batch and CECF were compared and in CECF reactions a higher yield of 250 mg/L IgG and 500 mg/L scFv-Fc were produced. From this total yield only a fraction (9 mg/L IgG; 36 mg/L scFv-Fc) was intact (Stech et al., 2017). A cell-free system for the synthesis of IgGs, based on CHO, was also developed by Martin et al. The reaction was supplemented with reduced and oxidized glutathione, DsbC and yPDI. As previously described, initiating synthesis of the light chain prior to addition of the heavy chain template enhanced the production of correctly folded, non-aggregated antibodies, resulting in yields exceeding 100 mg/L (Martin et al., 2017). Nanobodies were produced for the first time in a CHO-based cell-free system by Haueis et al., achieving yields of up to 12 mg/L (Haueis et al., 2022).

Due to their open and flexible nature, cell-free systems can be readily tailored for specialized applications, including the synthesis of antibody-drug conjugates and site-specific incorporation of non-canonical amino acids. A major advantage of these systems is their capacity for co-translational protein labeling. This platform allows the expression of cytotoxic proteins and the incorporation of pre-charged suppressor tRNAs or orthogonal tRNA/synthetase pairs. A commonly used strategy exploits stop codons for the site-specific insertion of non-natural amino acids, enabling the production of homogeneous conjugates.

The incorporation of non-canonical amino acids into a scFv was shown by Patel et al. In an *E. coli* cell-free system, a scFv and *Guassia princeps* luciferase were expressed separately and later conjugated by azide-alkyne click chemistry. The activity of the scFv fragment was retained (Patel et al., 2009). Site specific fluorescent labeling was shown for scFv and Fab Fragments in *E. coli* and for scFv and IgG in a CHO-based cell-free system (Stech et al., 2014; Stech et al., 2017; Matsuda et al., 2018).

Site specific labeling can also be used for the conjugation of antibodies with drugs. The conjugation of the IgG trastuzumab with a DBCO-PEG-monomethyl auristatin resulted in a highly potent ADC as shown in *in vitro* assays. The ADC was produced with a yield of 250 mg/L in an *E. coli*-based cell-free system (Zimmerman et al., 2014). A similar approach was conducted by Axup et al. trastuzumab IgG was site-specifically labeled with an auristatine derivative, exhibiting potent *in vitro* cytotoxic activity and complete tumor regression in a rodent model (Axup et al., 2012). As the incorporation site of the non-natural amino acid is critical for protein function, yield and following conjugation a workflow for the assessment of the incorporation position was established in a CHO-based cell-free system. The dual reporter system screening for activity and incorporation efficiency was tested with a scFv (Krebs et al., 2022a).

For the first time an antibody toxin conjugate was produced in a CHO and *E. coli* cell-free system by Krebs et al. An anti-CD7 scFv was conjugated with the toxin domain PE24, a shortened variant of *Pseudomonas* exotoxin A, directing the killing of CD7 positive cells (Krebs et al., 2022b). Here, the use of cell free systems enabled the production of immunotoxins that would otherwise kill their production cells.

In addition to their therapeutic applications, ADCs can be employed as vaccines to elicit targeted immune responses. A scFv against mouse B-cell lymphoma was linked to the cytokine GM-CSF and produced in an *E. coli* cell-free system. The efficacy of the tumor-specific humoral immune responses was comparable to mammalian produced whole immunoglobulin coupled to KLH (Kanter et al., 2007). In another study a scFv against mouse B-cell lymphoma was produced as a Tetanus Toxin Fragment C (TTFrC) fusion protein. The vaccine could protect from tumor challenge in a mouse model, 4 mg/L of purified vaccine were generated from an *E. coli*-based system (Patel et al., 2011).

Cell-free systems offer a wide range of additional applications in the production of antibody fragments. For example, they can be used for isotope labeling of antibodies, facilitating high-resolution NMR studies and Immuno-PET imaging (Matsuda et al., 2012; Giraud et al., 2024). Furthermore, scFv-functionalized therapeutic nanoparticles can be synthesized in a single-pot reaction, and both mono- and bispecific lipid nanoparticles have been successfully employed for mRNA delivery to T cells (Peruzzi et al., 2023). In another application, bispecific ‘knobs-into-holes’ antibodies were produced in an *E. coli*-based cell-free system, achieving yields exceeding 900 mg/L (Xu et al., 2015).

## 6 Human production *in vivo*

As described in this review, the recombinant production of proteins is complex and often associated with high production and purification costs. An ideal way to circumvent the tedious production of recombinant proteins for therapy in bioreactors would be the expression directly in the human body. BioNTech used lipid nanoparticle (LNP) encapsulated mRNA to produce bispecific antibodies *in vivo* against ovarian carcinoma and tested this approach in mice, resulting in the elimination of the tumor (Stadler et al., 2017). A further important step by CureVac was the *in vivo* production of an antibody against rabies virus and, respectively, against botulinum toxin A (BotA) in mice, showing a protection against the virus and toxin in both prophylactic and therapeutic challenge experiments (Thran et al., 2017). A breakthrough was the first clinical trial by Moderna. Kose et al. (2019) described this approach in 2019 for antibodies against chikungunya virus (CHIKV). In a first mice experiment, mRNA, encoding the neutralizing antibody CHKV-24, encapsulated in LNPs was injected intravenously and subsequently the mice were challenged with a lethal dose of CHIKV. This resulted in a dose-dependent survival of the mice. In an experiment with macaques, serum titers up to 28.8 mg/L were reached and the antibodies were still detected 90 days after mRNA administration. In a clinical phase 1 trial (NCT03829384) the production of CHKV-24 was tested in humans (August et al., 2021). Depending on the mRNA dose (0.1, 0.3, 0.6 mg/kg or 2 × 0.3 mg/kg), 2–10 mg/L IgG were produced after 36–48 h. The antibody concentration was above 1 mg/L for over 16 weeks for the 0.3 and 0.6 mg/kg group. The serum after 12, 24 and 48 h was used for *ex vivo* CHIKV neutralization experiments (plaque reduction assay) and showed a very good neutralization for the groups who received 0.3 or 0.6 mg/kg for all three time points. This first approach with LNP-encapsulated mRNA encoding an anti-CHIKV antibody and the corresponding clinical trial was the blueprint for further developments in this field. Similar approaches were published for influenza A virus (Van Hoecke et al., 2020) and SARS-CoV-2 (Deng et al., 2022) in the field of anti-viral antibodies. In the cancer field, this approach was also used for the expression of bispecifics to reduce the growth of EGFR-positive tumors (Hangiu et al., 2024), the expression of the anti-PD-1 antibody pembrolizumab (Wu L et al., 2022) or anti-HER2 antibody trastuzumab (Rybakova et al., 2019). An approach of mRNA delivery without LNPs by application of pure mRNA solutions as intratracheal aerosol was demonstrated for the anti-respiratory syncytial virus (RSV) antibody palivizumab and anti-RSV antibody fragments. Here, authors have shown for the most antibody constructs a reduced viral load *in vitro* and *in vivo* (Tiwari et al., 2018).

In summary, the approach using LNP-encapsulated mRNA to produce antibodies directly in humans could reduce the development and production costs and more importantly it could significantly accelerate the drug development, as also shown for mRNA vaccines during the COVID-19 pandemic. The successful development of COVID-19 vaccines was built on the prior experience of companies like Moderna, CureVac, and BioNTech, which had already established robust mRNA delivery systems for therapeutic antibodies well before the pandemic. We can expect several new antibody mRNA-based drugs in the future.

## 7 Concluding remarks

When we look at the production systems for EMA/FDA-approved antibodies, it becomes clear that the vast majority of them are manufactured using mammalian cells (Supplementary Table 1). Of 157 antibody therapeutics based on soluble products (CAR-T cells are not included, since their antibody portions are all produced *in vivo* in the patient’s own cells), 148 (94%) are made by recombinant production in rodent cells. Among these, 119 are produced in CHO cells (76%), further highlighting the clear dominance of this system in therapeutic antibody manufacturing, 29 (18%) in murine or rat myeloma cell systems (18 in murine NS0, 7 in murine Sp2/0, one each in murine A6(H4C5) and rat YB2/0 cells and two in unspecified myeloma cells), one full IgG in the yeast *Komagataella phaffii* (known as *Pichia pastoris* before 2009) and six (4%) in *E. coli*, which are all smaller fragments.^2^ This is despite robust bioprocesses for the production of recombinant antibodies in various organisms are well established and offered by a large number of commercial suppliers. However, their adaptation to specific applications is not determined solely by scientific factors. In the case of therapeutic antibodies, the introduction of a new production system or host organism represents a significant regulatory risk factor for the early development of a drug. Often, this alone leads to the decision to stick with the most conservative solution (CHO cells). However, beyond the conservative selection strategy of pharmaceutical companies developing therapeutics, antibodies can now be obtained from a variety of expression systems based on many different organisms from various systematic kingdoms. While the selection of a particular recombinant antibody production system is usually determined by availability, ease-of-use and cost-per-yield, other factors such as antibody format (IgG, scFv, Fab, bispecific antibodies, etc.) and correct post-translational modifications (e.g., glycosylation) must also be taken into account. For example, production in *E. coli* is already widely used for the production of smaller antibody fragments (scFv, Fab) due to easier handling, lower cost and widespread experience with this organism, but IgG cannot be made this way. To produce full immunoglobulins, the yields that can be achieved with the widely used and ‘kit-based’ mammalian cell systems are often sufficient for laboratory applications or diagnostic tests, so there is little motivation to seek alternatives for the use of serum-free approaches, or other expression systems. Furthermore, most other expression systems require novel cultivation techniques and media, additional administrative effort for applying for genetic engineering work, and additional general expertise on organisms that are often rather exotic for most laboratories. Nevertheless, several plant-based antibody drugs have reached clinical application, and the fundamental scientific questions behind a large variety of different expression systems have been resolved. It appears that the choice of production system today is determined more by yield/cost, local expertise, and regulatory factors than by a general lack of options. CHO cells continue to lead the way in terms of yield, but this may be due to the enormous optimization efforts that have been invested in this system over several decades (Jostock, 2011). Other systems might also achieve similar performance in the future with appropriate optimisation. In any case, various completely animal-free methods can already be used today for the discovery of antibodies with new specificities (Dübel, 2024). The variety of animal-free methods available today for the technical production of immunoglobulins proves that this step can now also be carried out completely “vegan” if desired.

## Footnotes

1
https://hfpappexternal.fda.gov/scripts/fdcc/index.cfm?set=GRASNotices (accessed 13. November 2025).

2 Antibody Society, https://www.antibodysociety.org/antibody-therapeutics-product-data/, EMA and FDA (as of 1/30/2026).
