# Supplementary material for: The landscape of antibody production systems: recombinant expression for research, diagnostics and therapy
Source: Front Bioeng Biotechnol. 2026 May 20;14:1765764. doi: 10.3389/fbioe.2026.1765764 (PMC13231048; doi:10.3389/fbioe.2026.1765764)
Supplement: Supplementary file 1 [file DataSheet1.pdf]

Supplemental Table 1: Production systems of approved therapeutic antibodies. Abbreviations, ADC, antibody drug conjugate, CHO, chinese hamster ovary; NSO: Murine myeloma non-secreting 0, RIC, radioImmuno-conjugate, TBD, information not available. Source: Antibody Society, https://www.antibodysociety.org/antibody-therapeutics-product-data/, EMA and FDA (as of 1/30/2026).

**INN**	**Expression system**	**Format**	**Backbone**	**Light Chain**	**Specificity**	**Sequence source**	**Conjugated / unconjugated**	**Target**	**Indication First Approved or**	**First global approval**
[fam] Trastuzumab deruxtecan	CHO cells	Full-length antibody	IgG1	kappa	Monospecific	Humanized	ADC	HER2	HER2+ metastatic breast cancer	US, 2019
Adalimumab	CHO cells	Full-length antibody	IgG1	kappa	Monospecific	Human	Unconjugated	TNF	Rheumatoid arthritis	US, 2002
Ado-Trastuzumab emtansine	CHO cells	Full-length antibody	IgG1	kappa	Monospecific	Humanized	ADC	HER2	Breast cancer	US, 2013
Aducanumab	CHO cells	Full-length antibody	IgG1	kappa	Monospecific	Human	Unconjugated	Amyloid beta	Alzheimer's disease	US, 2021
Alemtuzumab	CHO cells	Full-length antibody	IgG1	kappa	Monospecific	Humanized	Unconjugated	CD52	Multiple sclerosis; chronic myeloid leukemia#	US, 2001
Alirocumab	CHO cells	Full-length antibody	IgG1	kappa	Monospecific	Human	Unconjugated	PCSK9	High cholesterol	US, 2015
Ansuvimab	CHO cells	Full-length antibody	IgG1	kappa	Monospecific	Human	Unconjugated	Ebola virus glycoprotein	Ebola virus infection	US, 2020
Atezolizumab	CHO cells	Full-length antibody	IgG1	kappa	Monospecific	Humanized	Unconjugated	PD-L1	Bladder cancer	US, 2016
Atoltivimab, Maftivimab, and Odesivimab	CHO cells	Full-length antibodies, mixture of 3	IgG1	kappa	Mixture of 3 monospecific antibodies	Human	Unconjugated	Ebola virus	Ebola virus infection	US, 2020
Avelumab	CHO cells	Full-length antibody	IgG1	lambda	Monospecific	Human	Unconjugated	PD-L1	Merkel cell carcinoma	US, 2017
Bevacizumab	CHO cells	Full-length antibody	IgG1	kappa	Monospecific	Humanized	Unconjugated	VEGF-A	Colorectal cancer	US, 2004
Bezlotoxumab	CHO cells	Full-length antibody	IgG1	kappa	Monospecific	Human	Unconjugated	Clostridium difficile enterotoxin B	Prevention of Clostridium difficile infection recurrence	US, 2016
Bimekizumab	CHO cells	Full-length antibody	IgG1	kappa	Monospecific	Humanized	Unconjugated	IL-17A and IL-17F	Psoriasis	EU, 2021
Blinatumomab	CHO cells	Tandem scFv	Tandem scFv	NA	Bispecific	Murine	Unconjugated	CD19, CD3	Acute lymphoblastic leukemia	US, 2014

Brentuximab vedotin	CHO cells	Full-length antibody	IgG1	kappa	Monospecific	Chimeric mouse/ human	ADC	CD30	Hodgkin lymphoma, systemic anaplastic large cell lymphoma	US, 2011
Brodalumab	CHO cells	Full-length antibody	IgG2	kappa	Monospecific	Human	Unconjugated	IL-17R	Plaque psoriasis	Japan, 2016
Burosumab	CHO cells	Full-length antibody	IgG1	kappa	Monospecific	Human	Unconjugated	FGF23	X-linked hypophosphatemia	EU, 2018
Casirivimab + Imdevimab	CHO cells	Full-length antibodies, mixture of 2	IgG1	kappa and lambda	Mixture of 2 monospecific antibodies	Human	Unconjugated	SARS-CoV-2	COVID-19	Japan, 2021
Cemiplimab	CHO cells	Full-length antibody	IgG4	kappa	Monospecific	Human	Unconjugated	PD-1	squamous cell skin cancer	US, 2018
Concizumab	CHO cells	Full-length antibody	IgG4	kappa	Monospecific	Humanized	Unconjugated	Tissue factor pathway inhibitor	Hemophilia A or B	Canada, 2023
Cosibelimab,	CHO cells	Full-length antibody	IgG1	lambda	Monospecific	Human	Unconjugated	PD-L1	Squamous cell carcinoma	US, 2024
Crizanlizumab	CHO cells	Full-length antibody	IgG2	kappa	Monospecific	Humanized	Unconjugated	CD62 (P-selectin)	Sickle cell disease	US, 2019
Crovalimab	CHO cells	Full-length antibody	IgG1	kappa	Monospecific	Humanized	Unconjugated	Complement C5	Atypical hemolytic uremic syndrome	China, 2024
Daratumumab	CHO cells	Full-length antibody	IgG1	kappa	Monospecific	Human	Unconjugated	CD38	Multiple myeloma	US, 2015
Denosumab	CHO cells	Full-length antibody	IgG2	kappa	Monospecific	Human	Unconjugated	RANK-L	Bone Loss	EU, 2010
Donanemab	CHO cells	Full-length antibody	IgG1	kappa	Monospecific	Humanized	Unconjugated	Amyloid beta, N3pG (N-terminal truncated)	Alzheimer's disease	US, 2024
Dostarlimab	CHO cells	Full-length antibody	IgG4	kappa	Monospecific	Humanized	Unconjugated	PD-1	Endometrial cancer	EU, 2021
Dupilumab	CHO cells	Full-length antibody	IgG4	kappa	Monospecific	Human	Unconjugated	IL-4Rα	Atopic dermatitis	US, 2017
Durvalumab	CHO cells	Full-length antibody	IgG1	kappa	Monospecific	Human	Unconjugated	PD-L1	Bladder cancer	US, 2017
Efalizumab	CHO cells	Full-length antibody	IgG1	kappa	Monospecific	Humanized	Unconjugated	CD11a	Psoriasis	US, 2003
Elranatamab	CHO cells	Full-length antibody	IgG2	kappa	Bispecific	Humanized	Unconjugated	BCMA, CD3	Multiple myeloma	US, 2023

Emapalumab	CHO cells	Full-length antibody	IgG1	lambda	Monospecific	Human	Unconjugated	IFNg	Primary hemophagocytic lymphohistiocytosis	US, 2018
Emicizumab	CHO cells	Full-length antibody	IgG4	kappa	Bispecific	Humanized	Unconjugated	Factor IXa, Factor X	Hemophilia A	US, 2017
Enfortumab vedotin	CHO cells	Full-length antibody	IgG1	kappa	Monospecific	Human	ADC	Nectin-4	Urothelial cancer	US, 2019
Enlonstobart	CHO cells	Full-length antibody	IgG4	kappa	Monospecific	Human	Unconjugated	PD-1	Cervical cancer	China, 2024
Epcoritamab	CHO cells	Full-length antibody	IgG1; Hetero H	lambda/ kappa	Bispecific	Humanized	Unconjugated	CD20, CD3	Diffuse large B-cell lymphoma	US, 2023
Erenumab	CHO cells	Full-length antibody	IgG2	lambda	Monospecific	Human	Unconjugated	CGRP receptor	Migraine prevention	US, 2018
Evinacumab	CHO cells	Full-length antibody	IgG4	kappa	Monospecific	Human	Unconjugated	Angiopoietin-like 3	Homozygous familial hypercholesterolemia	US, 2021
Evolocumab	CHO cells	Full-length antibody	IgG2	lambda	Monospecific	Human	Unconjugated	PCSK9	High cholesterol	EU, 2015
Faricimab,	CHO cells	Full-length antibody	IgG1	kappa/ lambda	Bispecific	Humanized/ human	Unconjugated	VEGF-A, Ang-2	Neovascular age-related macular degeneration, diabetic macular edema	US, 2022
Fremanezumab	CHO cells	Full-length antibody	IgG2Δa	kappa	Monospecific	Humanized	Unconjugated	CGRP	Migraine prevention	US, 2018
Galcanezumab	CHO cells	Full-length antibody	IgG4	kappa	Monospecific	Humanized	Unconjugated	CGRP	Migraine prevention	US, 2018
Glofitamab	CHO cells	Fab-Fc x Fab-Fab-Fc	IgG1; CrossMab	lambda/ kappa	Bispecific	Humanized	Unconjugated	CD20, CD3e	Diffuse large B-cell lymphoma	Canada, 2023
Guselkumab	CHO cells	Full-length antibody	IgG1	lambda	Monospecific	Human	Unconjugated	IL-23 p19	Plaque psoriasis	US, 2017
Ibritumomab tiuxetan	CHO cells	Full-length antibody	mIgG1	kappa	Monospecific	Murine	RIC	CD20	Non-Hodgkin lymphoma	US, 2002
Idarucizumab	CHO cells	Fab	Fab (CH1 IgG1)	kappa	Monospecific	Humanized	Unconjugated	Dabigatran	Reversal of dabigatran-induced anticoagulation	US, 2015
Inotuzumab ozogamicin	CHO cells	Full-length antibody	IgG4	kappa	Monospecific	Humanized	ADC	CD22	Acute lymphoblastic leukemia	US, 2017
Ipilimumab	CHO cells	Full-length antibody	IgG1	kappa	Monospecific	Human	Unconjugated	CTLA-4	Metastatic melanoma	US, 2011

Isatuximab	CHO cells	Full-length antibody	IgG1	kappa	Monospecific	Chimeric mouse/ human	Unconjugated	CD38	Multiple myeloma	US, 2020
Ixekizumab	CHO cells	Full-length antibody	IgG4	kappa	Monospecific	Humanized	Unconjugated	IL-17A	Psoriasis	US, 2016
Lanadelumab	CHO cells	Full-length antibody	IgG1	kappa	Monospecific	Human	Unconjugated	Plasma kallikrein	Hereditary angioedema	US, 2018
Lebrikizumab	CHO cells	Full-length antibody	IgG4	kappa	Monospecific	Humanized	Unconjugated	IL-13	Atopic dermatitis	EU, 2023
Lecanemab	CHO cells	Full-length antibody	IgG1	kappa	Monospecific	Humanized	Unconjugated	Amyloid beta protofibrils	Alzheimer's disease	US, 2023
Loncastuximab tesirine	CHO cells	Full-length antibody	IgG1	kappa	Monospecific	Humanized	ADC	CD19	Diffuse large B-cell lymphoma	US, 2021
Margetuximab	CHO cells	Full-length antibody	IgG1	kappa	Monospecific	Chimeric mouse/ human	Unconjugated	HER2	HER2+ metastatic breast cancer	US, 2020
Mepolizumab	CHO cells	Full-length antibody	IgG1	kappa	Monospecific	Humanized	Unconjugated	IL-5	Severe eosinophilic asthma	US, 2015
Mirikizumab	CHO cells	Full-length antibody	IgG4	kappa	Monospecific	Humanized	Unconjugated	IL-23p19	Ulcerative colitis	Japan, 2023
Mirvetuximab soravtansine	CHO cells	Full-length antibody	IgG1	kappa	Monospecific	Humanized	ADC	FR	Ovarian cancer	US, 2022
Mosunetuzumab	CHO cells	Full-length antibody	IgG1; Hetero H, HL assembly	kappa	Bispecific	Humanized	Unconjugated	CD20, CD3	Follicular lymphoma	EU, 2022
Naxitamab	CHO cells	Full-length antibody	IgG1	kappa	Monospecific	Humanized	Unconjugated	GD2	High-risk neuroblastoma and refractory osteomedullary disease	US, 2020
Nemolizumab	CHO cells	Full-length antibody	IgG2	kappa	Monospecific	Humanized	Unconjugated	IL-31R	Pruritus with atopic dermatitis	Japan, 2022
Nirsevimab	CHO cells	Full-length antibody	IgG1	kappa	Monospecific	Human	Unconjugated	RSV	Prevention of respiratory syncytial virus infection	EU, 2022
Nivolumab	CHO cells	Full-length antibody	IgG4	kappa	Monospecific	Human	Unconjugated	PD-1	Melanoma, non-small cell lung cancer	US, 2014
Ocrelizumab	CHO cells	Full-length antibody	IgG1	kappa	Monospecific	Humanized	Unconjugated	CD20	Multiple sclerosis	US, 2017

Omalizumab	CHO cells	Full-length antibody	IgG1	kappa	Monospecific	Humanized	Unconjugated	IgE	Asthma	US, 2003
Panitumumab	CHO cells	Full-length antibody	IgG2	kappa	Monospecific	Human	Unconjugated	EGFR	Colorectal cancer	US, 2006
Pembrolizumab	CHO cells	Full-length antibody	IgG4	kappa	Monospecific	Humanized	Unconjugated	PD-1	Melanoma	US, 2014
Pertuzumab	CHO cells	Full-length antibody	IgG1	kappa	Monospecific	Humanized	Unconjugated	HER2	Breast Cancer	US, 2012
Polatuzumab vedotin	CHO cells	Full-length antibody	IgG1	kappa	Monospecific	Humanized	ADC	CD79b	Diffuse large B-cell lymphoma	US, 2019
Pozelimab	CHO cells	Full-length antibody	IgG4	kappa	Monospecific	Human	Unconjugated	Complement C5	CHAPLE disease	US, 2023
Ravulizumab	CHO cells	Full-length antibody	IgG2(CH1-hinge)/ IgG4(CH2-CH3)	kappa	Monospecific	Humanized	Unconjugated	Complement C5	Paroxysmal nocturnal hemoglobinuria	US, 2018
Relatlimab	CHO cells	Full-length antibody	IgG4	kappa	Monospecific	Human	Unconjugated	LAG-3	Melanoma	US, 2022
Retifanlimab	CHO cells	Full-length antibody	IgG4	kappa	Monospecific	Humanized	Unconjugated	PD-1	Merkel cell carcinoma	US, 2023
Risankizumab	CHO cells	Full-length antibody	IgG1	kappa	Monospecific	Humanized	Unconjugated	IL-23 p19	Plaque psoriasis	Japan, 2019
Rituximab	CHO cells	Full-length antibody	IgG1	kappa	Monospecific	Chimeric mouse/ human	Unconjugated	CD20	Non-Hodgkin lymphoma	US, 1997
Romosozumab	CHO cells	Full-length antibody	IgG2	kappa	Monospecific	Humanized	Unconjugated	Sclerostin	Osteoporosis in postmenopausal women at increased risk of fracture	Japan, 2019
Sarilumab	CHO cells	Full-length antibody	IgG1	kappa	Monospecific	Human	Unconjugated	IL-6R	Rheumatoid arthritis	Canada, 2017
Satralizumab	CHO cells	Full-length antibody	IgG2	kappa	Monospecific	Humanized	Unconjugated	IL-6R	Neuromyelitis optica spectrum disorder	Canada, 2020
Secukinumab	CHO cells	Full-length antibody	IgG1	kappa	Monospecific	Human	Unconjugated	IL-17A	Psoriasis	Japan, 2014
Siltuximab	CHO cells	Full-length antibody	IgG1	kappa	Monospecific	Chimeric mouse/ human	Unconjugated	IL-6	Castleman disease	US, 2014
Sotrovimab	CHO cells	Full-length antibody	IgG1	kappa	Monospecific	Human	Unconjugated	SARS-CoV-2	COVID-19	Australia, 2021

Spesolimab	CHO cells	Full-length antibody	IgG1	kappa	Monospecific	Humanized	Unconjugated	IL-36R	Generalized pustular psoriasis	US, 2022
Sugemalimab	CHO cells	Full-length antibody	IgG4	lambda	Monospecific	Human	Unconjugated	PD-L1	Non-small cell lung cancer	China, 2021
Sutimlimab	CHO cells	Full-length antibody	IgG4	kappa	Monospecific	Humanized	Unconjugated	Complement C1s	Cold agglutinin disease	US, 2022
Tafasitamab	CHO cells	Full-length antibody	IgG1/2 hybrid	kappa	Monospecific	Humanized	Unconjugated	CD19	Diffuse large B-cell lymphoma	US, 2020
Talquetamab	CHO cells	Full-length antibody	IgG4	lambda/ kappa	Bispecific	Humanized	Unconjugated	GPCR5D, CD3	Multiple myeloma	US, 2023
Teclistamab	CHO cells	Full-length antibody	IgG4; Hetero H, HL exchanged	lambda	Bispecific	Humanized	Unconjugated	BCMA, CD3	Multiple myeloma	EU, 2022
plizumab	CHO cells	Full-length antibody	IgG1	kappa	Monospecific	Humanized	Unconjugated	CD3	Type 1 diabetes	US, 2022
Teprotumumab	CHO cells	Full-length antibody	IgG1	kappa	Monospecific	Human	Unconjugated	IGF-1R	Thyroid eye disease	US, 2020
Tezepelumab	CHO cells	Full-length antibody	IgG2	lambda	Monospecific	Human	Unconjugated	Thymic stromal lymphopoietin	Severe asthma	US, 2021
Tildrakizumab	CHO cells	Full-length antibody	IgG1	kappa	Monospecific	Humanized	Unconjugated	IL-23 p19	Plaque psoriasis	US, 2018
Tislelizumab	CHO cells	Full-length antibody	IgG4	kappa	Monospecific	Humanized	Unconjugated	PD-1	Esophageal squamous cell carcinoma	China, 2019
Tisotumab vedotin	CHO cells	Full-length antibody	IgG1	kappa	Monospecific	Human	ADC	Tissue factor	Cervical cancer	US, 2021
Tixagevimab, Cilgavimab	CHO cells	Full-length antibodies, mixture of 2	IgG1	kappa	Mixture of 2 monospecific antibodies	Human	Unconjugated	SARS-CoV-2	COVID-19	EU, 2022
Tocilizumab	CHO cells	Full-length antibody	IgG1	kappa	Monospecific	Humanized	Unconjugated	IL-6R	Rheumatoid arthritis	Japan, 2005
Toripalimab	CHO cells	Full-length antibody	IgG4	kappa	Monospecific	Humanized	Unconjugated	PD-1	Nasopharyngeal carcinoma	China, 2018
Trastuzumab	CHO cells	Full-length antibody	IgG1	kappa	Monospecific	Humanized	Unconjugated	HER2	Breast cancer	US, 1998
Vedolizumab	CHO cells	Full-length antibody	IgG1	kappa	Monospecific	Humanized	Unconjugated	α4β7 integrin	Ulcerative colitis, Crohn disease	US, 2014

Zolbetuximab	CHO cells	Full-length antibody	IgG1	kappa	Monospecific	Chimeric mouse/ human	Unconjugated	Claudin-18.2	HER2-negative gastric or gastroesophageal junction adenocarcinoma	Japan, 2024
Odronextamab	CHO cells	Full-length antibody	IgG4 Hetero H, cL	kappa	Bispecific	Human	Unconjugated	CD20, CD3	Relapsed/ refractory (R/R) follicular lymphoma or R/R diffuse large B-cell lymphoma	EU, 2024
Marstacimab	CHO cells	Full-length antibody	IgG1	lambda	Monospecific	Human	Unconjugated	Tissue factor pathway inhibitor	Hemophilia	US, 2024
Tarlatamab	CHO cells	scFv-scFv-scFc	TBD	TBD	Bispecific	TBD	Unconjugated	DLL3, CD3	Small cell lung cancer	US, 2024
Axatilimab	CHO cells	Full-length antibody	IgG4	kappa	Monospecific	Humanized	Unconjugated	CSF-1R	Graft vs. host disease	US, 2024
Zanidatamab	CHO cells	scFv-Fc x Fab-Fc	TBD	TBD	Bispecific	Humanized	Unconjugated	HER2, HER2 (biparatopic)	Biliary tract cancers	US, 2024
Datopotamab deruxtecan	CHO cells	Full-length antibody	IgG1	kappa	Monospecific	Humanized	ADC	TROP-2	Breast cancer (HR+, HER2-)	Japan, 2024
Zenocutuzumab	CHO cells	Full-length antibody	IgG1	kappa	Bispecific	Humanized	Unconjugated	HER2, HER3	non-small cell lung cancer or pancreatic cancer	US, 2024
Vilobelimab	CHO cells	Full-length antibody	IgG4	kappa	Monospecific	Chimeric mouse/ human	Unconjugated	Complement C5a	SARS-CoV-2 induced septic acute respiratory distress syndrome	EU, 2025
Garadacimab	CHO cells	Full-length antibody	IgG4	lambda	Monospecific	Human	Unconjugated	Factor XIIa	Prevention of hereditary angioedema aoacks	Australia, 2025
Narlumosbart	CHO cells, cell line CHO-S, glycoform alfa	Full-length antibody	IgG4	kappa	Monospecific	Human	Unconjugated	RANK-L	Giant cell tumor of bone	China, 2023
Obinutuzumab	CHO cells, Coexpression with GnT III and α-ManII	Full-length antibody	IgG1	kappa	Monospecific	Humanized	Unconjugated	CD20	Chronic lymphocytic leukemia	US, 2013
Rozanolixizumab	CHO cells, DG44	Full-length antibody	IgG4	kappa	Monospecific	Humanized	Unconjugated	FcRn	Generalized myasthenia gravis	US, 2023

Belantamab mafodotin	CHO cells, FUT8−/− CHO cells	Full-length antibody	IgG1	kappa	Monospecific	Humanized	ADC	BCMA	Multiple myeloma	US, 2020
Benralizumab	CHO cells, FUT8−/− CHO cells	Full-length antibody	IgG1	kappa	Monospecific	Humanized	Unconjugated	IL-5R α	Asthma	US, 2017
Inebilizumab)	CHO cells, FUT8−/− CHO cells	Full-length antibody	IgG1	kappa	Monospecific	Humanized	Unconjugated	CD19	Neuromyelitis optica spectrum disorders	US, 2020
Mogamulizumab	CHO cells, FUT8−/− CHO cells	Full-length antibody	IgG1	kappa	Monospecific	Humanized	Unconjugated	CCR4	Mycosis fungoides or Sézary syndrome	Japan, 2012
Amivantamab	CHO cells, Low-fucose production cells	Full-length antibody	IgG1	kappa	Bispecific	Human	Unconjugated	EGFR, cMET	NSCLC w/ EGFR exon 20 insertion mutations	US, 2021
Ormutivimab	CHO-K1 cells	Full-length antibody	IgG1	lambda 2	Monospecific	Human	Unconjugated	Rabies virus surface glycoprotein 4	Post-exposure prophylaxis of rabies	China, 2022
Regdanvimab	CHO-K1 cells	Full-length antibody	IgG1	lambda	Monospecific	Human	Unconjugated	SARS-CoV-2	COVID-19	Republic of Korea, 2021
Nebacumab	Heteromyeloma cell line A6(H4C5)	Full-length antibody	IgM	kappa	Monospecific	Human	Unconjugated	Endotoxin	Gram-negative sepsis	Netherlands, England, France, Germany, 1991
Muromonab-CD3	Murine hybridoma	Full-length antibody	mIgG2a	kappa	Monospecific	Murine	Unconjugated	CD3	Reversal of kidney transplant rejection	US, 1986
Tositumomab-I131	Murine hybridoma	Full-length antibody	mIgG2a	lambda	Monospecific	Murine	RIC	CD20	Non-Hodgkin Lymphoma	US, 2003
Tralokinumab	Murine myeloma cells	Full-length antibody	IgG4	lambda	Monospecific	Human	Unconjugated	IL-13	Atopic dermatitis	EU, 2021
Sacituzumab govitecan	Murine myeloma cells	Full-length antibody	IgG1	kappa	Monospecific	Humanized	ADC	TROP-2	Triple-neg. breast cancer	US, 2020
Abciximab	Murine myeloma cells Sp2/0	Fab	Fab (CH1 IgG1)	kappa	Monospecific	Chimeric mouse/ human	Unconjugated	GPIIb/IIIa	Prevention of blood clots in angioplasty	US, 1994
Canakinumab	Murine myeloma cells Sp2/0	Full-length antibody	IgG1	kappa	Monospecific	Human	Unconjugated	IL-1β	Muckle-Wells syndrome	US, 2009

Cetuximab	Murine myeloma cells Sp2/0	Full-length antibody	IgG1	kappa	Monospecific	Chimeric mouse/ human	Unconjugated	EGFR	Colorectal cancer	Switzerland, 2003
Dinutuximab	Murine myeloma cells Sp2/0	Full-length antibody	IgG1	kappa	Monospecific	Chimeric mouse/ human	Unconjugated	GD2	Neuroblastoma	US, 2015
Golimumab	Murine myeloma cells Sp2/0	Full-length antibody	IgG1	kappa	Monospecific	Human	Unconjugated	TNF	Rheumatoid and psoriatic arthritis, ankylosing spondylitis	US, 2009
Infliximab	Murine myeloma cells Sp2/0	Full-length antibody	IgG1	kappa	Monospecific	Chimeric mouse/ human	Unconjugated	TNF	Crohn disease	US, 1998
Ustekinumab	Murine myeloma cells Sp2/0	Full-length antibody	IgG1	kappa	Monospecific	Human	Unconjugated	IL-12/23	Psoriasis	EU, 2009
Anifrolumab	NSO cells	Full-length antibody	IgG1	kappa	Monospecific	Human	Unconjugated	IFNAR1	Systemic lupus erythematosus	US, 2021
Obiltoxaximab	NSO cells	Full-length antibody	IgG1	kappa	Monospecific	Chimeric mouse/ human	Unconjugated	*B. anthrasis* PA	Prevention of inhalational anthrax	US, 2016
Basiliximab	NSO cells	Full-length antibody	IgG1	kappa	Monospecific	Chimeric mouse/ human	Unconjugated	IL-2R	Prevention of kidney transplant rejection	US, 1998
Belimumab	NSO cells	Full-length antibody	IgG1	lambda	Monospecific	Human	Unconjugated	BLyS	Systemic lupus erythematosus	US, 2011
Daclizumab	NSO cells	Full-length antibody	IgG1	kappa	Monospecific	Humanized	Unconjugated	CD25	Multiple sclerosis; prevention of kidney transplant rejection	US, 1997
Eculizumab	NSO cells	Full-length antibody	gG2(CH1-hinge)/IgG4(CH2-CH3)	kappa	Monospecific	Humanized	Unconjugated	Complement C5	Paroxysmal nocturnal hemoglobinuria	US, 2007
Elotuzumab	NSO cells	Full-length antibody	IgG1	kappa	Monospecific	Humanized	Unconjugated	SLAMF7	Multiple myeloma	US, 2015
Gemtuzumab ozogamicin	NSO cells	Full-length antibody	IgG4	kappa	Monospecific	Humanized	ADC	CD33	Acute myeloid leukemia	US, 2000
Ibalizumab	NSO cells	Full-length antibody	IgG4	kappa	Monospecific	Humanized	Unconjugated	CD4	HIV infection	US, 2018
Natalizumab	NSO cells	Full-length antibody	IgG4	kappa	Monospecific	Humanized	Unconjugated	a4 integrin	Multiple sclerosis	US, 2004

Necitumumab	NSO cells	Full-length antibody	IgG1	kappa	Monospecific	Human	Unconjugated	EGFR	Non-small cell lung cancer	US, 2015
Ofatumumab	NSO cells	Full-length antibody	IgG1	kappa	Monospecific	Human	Unconjugated	CD20	Chronic lymphocytic leukemia	US, 2009
Olaratumab	NSO cells	Full-length antibody	IgG1	kappa	Monospecific	Human	Unconjugated	PDGFRα	Soft tissue sarcoma	US, 2016
Palivizumab	NSO cells	Full-length antibody	IgG1	kappa	Monospecific	Humanized	Unconjugated	RSV	Prevention of respiratory syncytial virus infection	US, 1998
Ramucirumab	NSO cells	Full-length antibody	IgG1	kappa	Monospecific	Human	Unconjugated	VEGFR2	Gastric cancer	US, 2014
Raxibacumab	NSO cells	Full-length antibody	IgG1	lambda	Monospecific	Human	Unconjugated	*B. anthracis* PA	Anthrax infection	US, 2012
Reslizumab	NSO cells	Full-length antibody	IgG4	kappa	Monospecific	Humanized	Unconjugated	IL-5	Asthma	US, 2016
Tremelimumab	NSO cells	Full-length antibody	IgG2	kappa	Monospecific	Human	Unconjugated	CTLA-4	Antineoplastic liver cancer	US, 2022
Ublituximab	Rat YB2/0 cells	Full-length antibody	IgG1	kappa	Monospecific	Chimeric mouse/ human	Unconjugated	CD20	Multiple sclerosis	US, 2022
Catumaxomab	Rat-mouse hybrid-hybridoma cell line	Full-length antibody	mIgG2a/k and rIgG2b/λ Hybrid	kappa/ lambda	Bispecific	Hybrid Rat/ Mouse	Unconjugated	EPCAM, CD3	Malignant ascites	EU, 2009
Eptinezumab	*Pichia pastoris* yeast cells *(Komagataella phaffii)*	Full-length antibody	IgG1	kappa	Monospecific	Humanized	Unconjugated	CGRP	Migraine prevention	US, 2020
Caplacizumab	*E. coli* bacteria	VHH (bivalent)	VHH (bivalent)	NA	Monospecific	Humanized	Unconjugated	von Willebrand factor	Acquired thrombotic thrombocytopenic purpura	EU, 2018
Certolizumab pegol	*E. coli* bacteria	PEGylated Fab	Fab (CH1 IgG1)	kappa	Monospecific	Humanized	PEGylated	TNF	Crohn disease	US, 2008
Moxetumomab pasudotox	*E. coli* bacteria	dsFv immunotoxin	dsFv fused with PE38 exotoxin		Monospecific	Murine	Immuno-conjugate	CD22	Hairy cell leukemia	US, 2018

Ranibizumab	*E. coli* bacteria	Fab	Fab (CH1 IgG1)	kappa	Monospecific	Humanized	Unconjugated	VEGF-A	Macular degeneration	US, 2006
Tebentafusp	*E. coli* bacteria	scFv-TCR fusion protein	scFv fused with a TCR	To be confirm ed	Bispecific	Humanized	Immuno-conjugate	gp100, CD3	Metastatic uveal melanoma	US, 2022
Brolucizumab	*E. coli* BL21(DE3) bacteria	scFv	scFv	kappa	Monospecific	Humanized	Unconjugated	VEGF-A	Neovascular age-related macular degeneration	US, 2019
